# Differences and similarities in the chemical bonding of intermetallic phases in the Ca–Al–Pt system

**DOI:** 10.1039/d5sc02993g

**Published:** 2025-08-28

**Authors:** Peter C. Müller, Linda S. Reitz, Stefan Engel, Richard Dronskowski, Oliver Janka

**Affiliations:** a Institute of Inorganic Chemistry, RWTH Aachen University, Jülich-Aachen Research Alliance (JARA-CSD), RWTH Aachen University 52056 Aachen Germany; b Inorganic Solid State Chemistry, Saarland University Campus C4.1 66123 Saarbrücken Germany oliver.janka@uni-saarland.de

## Abstract

Intermetallic compounds belong to an important class of materials, not only due to the sheer number of compounds known but also due to their application in everyday life. These compounds possess their very own peculiarities, especially when it comes to chemical bonding. To address this point, bonding analyses based on Crystal Orbital Bond Index (COBI) values, Löwdin charges, and – for the first time – *ab initio* oxidation numbers (ON^*ai*^) were conducted, all extracted from delocalized plane-wave functions. From the integrated COBI values, to be understood as quantum-chemical bond orders, the differences and similarities in the bonding behavior of the elements, binary and ternary compounds in the Ca–Al–Pt system were analyzed. It became apparent that the Al–Pt interactions, almost regardless of the respective compounds, show significant covalency, while Ca–Al and Ca–Pt interactions are of ionic nature in most cases. Homoatomic Al–Al or Pt–Pt interactions, however, tend to be ambivalent, depending on the respective crystal structure of a given compound.

## Abstract 2

The intermetallic Ca–Al–Pt system shows diverse features – structurally and quantum-chemically – that are examined using the Crystal Orbital Bond Index and Löwdin charges. This analysis is complemented by the newly introduced *ab initio* oxidation numbers.

## Graphical abstract

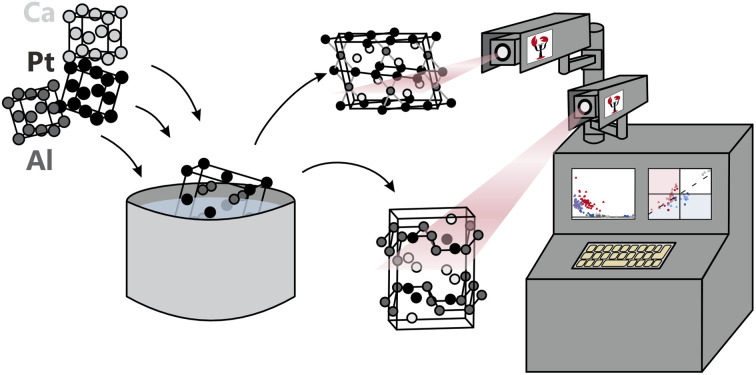

## 1. Introduction

Alloys and intermetallic compounds are important materials used in a manifold of modern everyday applications. Light-weight alloys, usually based on Be, Mg, Al, and Ti, are utilized in consumer goods, as well as in the transportation and construction sector.^1–7^ In addition, the most commonly used permanent magnets,^8,9^ materials with high thermal stability and corrosion resistance,^10–13^ or heterogeneous catalysts can be found amongst intermetallic compounds.^14–19^ They achieved practical use already way before their intrinsic characteristics were fully understood since they can often be obtained directly from the reaction of the constituent metals by metallurgical processes, arc-melting or metal fluxes.^20–22^ Nonetheless, the fundamental understanding of crystal chemistry, existence ranges and the chemical bonding in this class of materials is still ongoing.^23–27^ To quote Yuri Grin: “The main problem of chemists with intermetallic compounds is that they do not follow the usual valence rules. Therefore, for a longer time, these substances were not really considered as inorganic compounds”.^28^

Disobeying the classical valence rules leads to some problems because unless the underlying wave function is known and analyzed the nature of the chemical bond cannot be understood.^29–38^ What are the contributions to a certain interaction between two atoms? Ionic interactions or rather covalency? Does a concept of, say, electronegativity still apply? When going to the extremes, *e.g.*, Cs_2_Pt^39,40^ or CsAu,^40–42^ transparent compounds with salt-like behavior can be observed; however, when ΔEN is not as striking as in these examples, is there still a polarity in these intermetallic phases? The term ‘polar intermetallics’ is used quite frequently to describe compounds where a certain polarization within the structure is either assumed or proven. The question of how to properly address the bonding in solids dates back to almost a century: Laves^43^ and Pauling^44–46^ introduced atomic packing and “resonance” concepts for crystalline solids, while Hume-Rothery^47^ addressed the electronic (metallic) state and critical valence-electron concentrations.

Science has come a long way, since nowadays chemical bonding^48^ may be addressed computationally based on density-functional theory, either resting on the wave function^49–51^ or on the electron density.^52–56^ The main difference between both approaches lies in the examined quantity:^34^ a density-based method such as Bader's quantum theory of atoms in molecules (QTAIM) semi-classically partitions the electron density according to its topology. Even though this looks attractive since the density (1) is easily accessible from DFT calculations and (2) also experimentally observable from, *e.g.*, X-ray diffraction, the density lacks essential information, which is crucial for a well-grounded evaluation. As the density relates to the absolute square of the wave function, the phase information (*i.e.*, the sign of the wave function) is completely lost in the density, thus we cannot distinguish between bonding and antibonding, at least not from the wave function.

In a solid-state context, there is yet another hindrance: common DFT programs employ a delocalized plane-wave basis lacking local (chemical) information that would require atom-centered basis functions. In order to extract these bonding data, the program LOBSTER^57^ conducts a so-called projection from a plane-wave onto an atomic-orbital basis. Therefore, we regain chemistry in terms of, *e.g.*, Löwdin charges^58^ and crystal orbital bond index (COBI) values, the latter including the phase information.^59^ Both established tools allow for a bond classification in terms of ionicity and covalency. Additionally, in this recent contribution, we also introduce wave-function derived oxidation numbers that aim to resolve the schism between quantum-mechanical charges and empirical oxidation states.

**Fig. 1:**
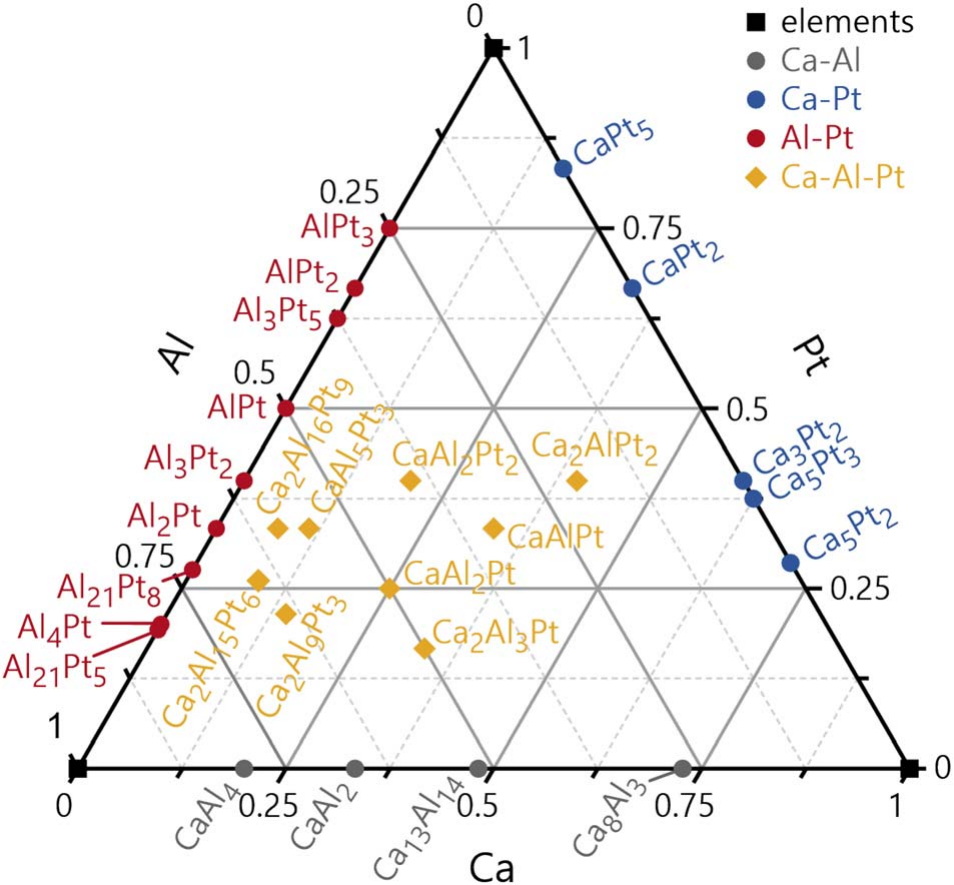
Gibbs triangle showing all reported phases in the ternary system Ca–Al–Pt (yellow). Element symbols are shown in black; binary compounds in the Ca–Al system are depicted in grey, in the Ca–Pt system in blue and in the Al–Pt system in red.

The case study in this paper will be the elements Ca, Al and Pt, selected binaries of the systems Ca–Al, Ca–Pt and Al–Pt, as well as all reported ternary compounds of the entire Ca–Al–Pt system. Fig. 1 depicts a Gibbs triangle showing all reported binary and ternary phases based on the Pearson database^60^ and the recent reports. Besides the crystal structures of the three elements Ca, Al and Pt,^61–63^ CaAl_2_ (ref. 64) and CaAl_4_,^64^ and CaPt_2_ (ref. 65) as well as Al_2_Pt,^66^ and AlPt^67^ were selected from the respective binary phase diagrams. Furthermore, CaAlPt, Ca_2_AlPt_2_ and CaAl_2_Pt were selected as representatives of ternary compounds. Our essential goal is to quantify the bonding nature in these intermetallic compounds, namely (1) to check if interatomic distances can be used as an identifier for bonding or non-bonding scenarios and (2) to identify similarities amongst the crystallographically significantly different compounds and highlight specific peculiarities that arise based on these quantum-chemical calculations.

## 2. Computational methods

Electronic structure calculations for the elements and selected binary as well as ternary compounds were performed using the projector augmented wave method (PAW) of Blöchl,^68^ as implemented in the Vienna *ab initio* simulation package (VASP).^69–73^ VASP calculations employed the Ca_sv, Al, Pt_pv, H and N pseudopotentials. The calculations started from the experimental crystallographic data but allowing for full structural optimizations, including lattice parameters and atomic positions. These were conducted for all compounds including electronic structure analyses. In all calculations, correlation and exchange were treated by the generalized gradient approximation of Perdew, Burke, and Ernzerhof (GGA-PBE).^74^ The cutoff energy for the plane-wave calculations was chosen to be a high value of 800 eV, and the convergence criteria for the energy differences between two iterative steps were set to 10^−6^ and 10^−5^ eV for the electronic and ionic steps, respectively. Brillouin zone integration was carried out using a *k*-point mesh with a spacing of ≈0.02 Å^−1^ for all compounds.

All corresponding electronic structures, based on the optimized calculations, were projected from plane waves onto a local orbital basis using the LOBSTER (Local Orbital Basis Suite Towards Electronic-structure Reconstruction) program package.^50,57,75–77^ By this method, the local density-of-states matrices (*i.e.*, energy-resolved wave-function eigenvectors) become available, which enable the calculation of the crystal orbital bond index (COBI),^59^ a generalized solid-state molecular bond index introduced by Wiberg^78^ and Mayer,^79^ as well as gross populations and atomic charges in a Löwdin-style formalism, in addition to *ab initio* oxidation numbers.^58^

## 3. Results and discussion

Before we proceed to analyze the chemical bonding, let us reiterate the underlying methods to describe a chemical bond. On a qualitatively correct level, (organic) chemists draw lines – “paired” electrons in a two-center two-electron bond – between two carbon atoms in order to symbolize an attractive C–C bond keeping these atoms together. Quantum-mechanically, this interaction can be described by a plethora of quantities. One of the most useful descriptors may be the bond index originally envisioned by Wiberg and Mayer that directly translates into the Lewis bond order, *i.e.*, 1 for a single bond, 2 for a double bond and so on. This bond index was then generalized to the solid state by means of the crystal orbital bond index (COBI) linking the Wiberg–Mayer bond idea to solid-state descriptors such as the crystal orbital overlap population by Hughbanks and Hoffmann^49^ and the crystal orbital Hamilton population by Dronskowski and Blöchl.^50,75^ The energy integral of the COBI, dubbed ICOBI, equals the bond order between two atoms in a solid. In molecular chemistry, integer bond orders prevail, but there are molecular exceptions such as a bond order of 1/2 in the hydrogen-molecule cation, H_2_^+^, or in the benzene aromatic C–C bond with a bond order of 3/2. In (inter-)metallic phases, however, fractional bond orders are the rule, typically characterized by ICOBI numbers well below the value of a single bond. This can be directly attributed to the larger coordination numbers found in condensed matter.

### 3.1. Oxidation numbers from quantum chemistry

Having introduced covalent bonding in solids, it is time to introduce a novel, yet related, method to calculate *ab initio* oxidation numbers (ON^*ai*^) such as to expand LOBSTER's quantum-chemical toolkit. All currently available population analyses homolytically split the bonding electrons, on purpose, and attribute half to each contributing atom: in a Mulliken-style scheme, this property is easily seen in the symmetric partitioning of the overlap population between two orbitals *μ* and *ν*. The Löwdin population analysis, however, uses an orthogonal basis, so an overlap population does not exist in the first place, but the homolytic splitting of bonding electrons is also utilized. This property becomes apparent when we consider the idempotency of the density matrix **P**, *i.e.*, Tr(**P**) = ½Tr(|**P**|^2^), and it lets us partition the electron density of any given compound into atom-centered electrons (½*P*_*μμ*_^2^) as well as bond-centered electrons (½|*P*_*μν*_|^2^). Just like Mulliken, Löwdin assigns the same number of bonding electrons to each atom without consideration of element-specific properties, such as electronegativity.

In the present work, we do address this issue, however, by introducing a weighting factor that allocates the bonding electrons to a more electronegative atom, in the spirit of heterolytic electron partitioning. This extension of the Löwdin population analysis then yields *ab initio* oxidation numbers ON^*ai*^ that are defined as the difference between the number of electrons in the neutral atom *N*_e,*A*_ and the population after heterolytic bond splitting:1
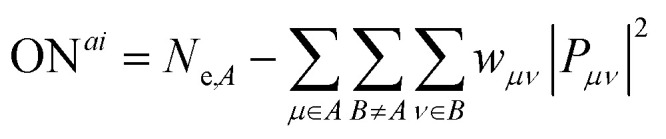


As the electronegativity is not straightforwardly accessible from quantum-mechanical calculations, we derive it by referring to the Hamilton matrix elements *H*_*μν*_ and constructing the weighting factor *w*_*μν*_:

2 *w*_*μν*_ = 1 + erf(*α*(*H*_*νν*_ − *H*_*μμ*_))

The error function and the parameter *α*††We note that the entire DFT procedure leading to the orbital picture is from first principles, without empirical parameters. To arrive at the chemists’ somewhat arbitrary heterolytical bond splitting, a likewise arbitrary parameter *α* cannot be avoided such as to not cause unreasonable shifts of bonding electrons. determine the “smearing” of the electronic partitioning between two atoms, and it ensures that minute energy differences between *H*_*μμ*_ and *H*_*νν*_ of, say, 0.1 eV as well as numerical noise do not cause an unreasonably drastic electron transfer. In the present study, we chose a value of *α* = 10 eV^−1^ which, for an energy difference of 0.1 eV, leads to the assignment of 92% electron density to the more electronegative atom and the remaining 8% to the less electronegative atom. A transfer of more than 99% is then performed at a difference of 0.165 eV.

**Fig. 2:**
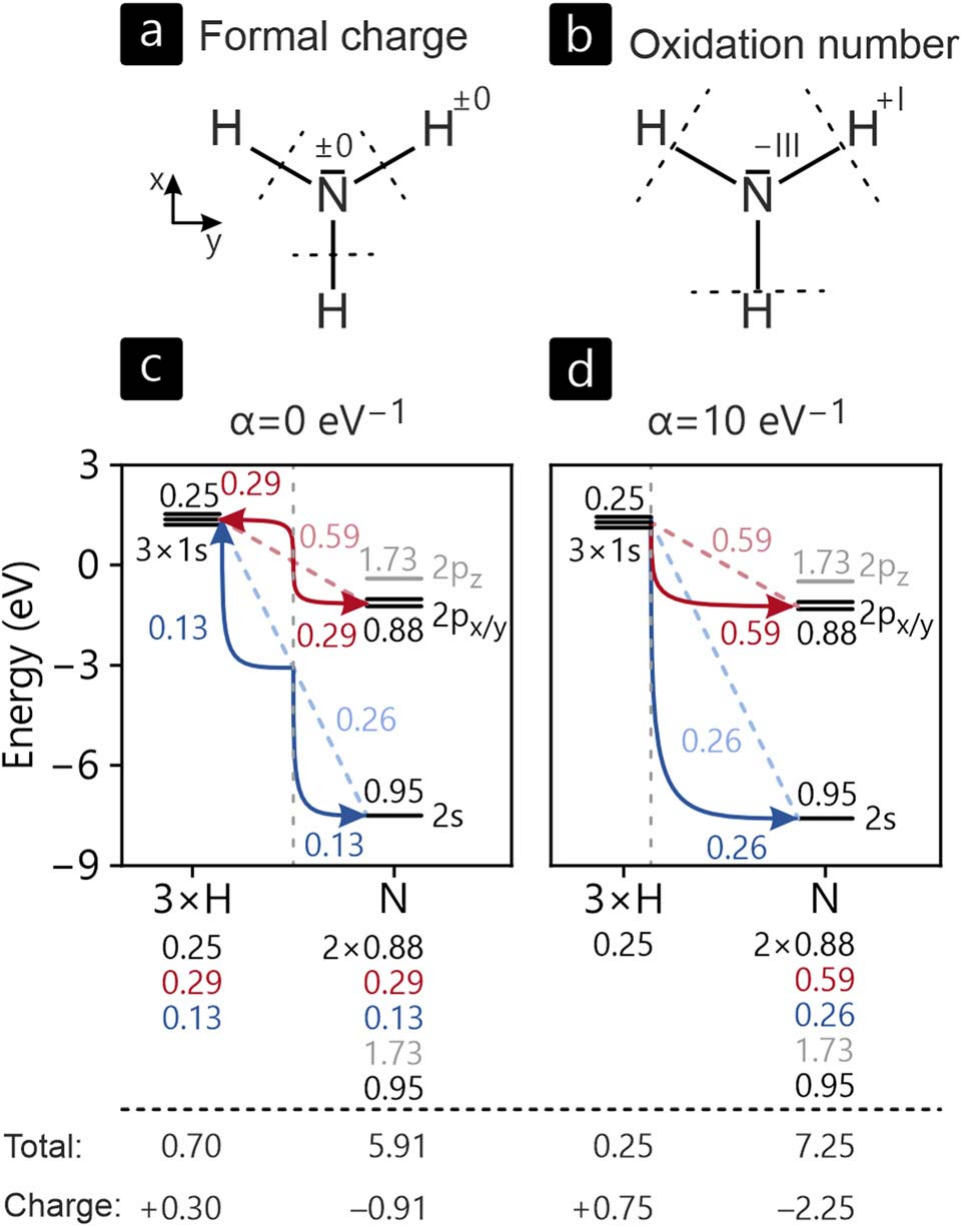
Algorithm to calculate (a and c) formal charges and (b and d) oxidation numbers from (a and b) empirical Lewis formulae and (c and d) *ab initio* population analyses for ammonia. Orbital and bond populations are given as numbers. A homolytic bond splitting, as done for the calculation of formal charges and *α* = 0, leads to the assignment of half the bonding electrons to each bonding partner. In the case of oxidation numbers, a heterolytic bond splitting is performed by *α* ≫ 0 such that all bonding electrons are assigned to the more electronegative atom.

The algorithm formulated in eqn (1) and (2) is visualized in Fig. 2 for the simple example of molecular ammonia, NH_3_. Fig. 2a shows the calculation of formal charges that result from a homolytic splitting of bonds. As such, the nitrogen atom is left with five electrons, and each hydrogen atom keeps one electron, leading to all atoms being formally neutral. When calculating oxidation numbers (*cf.*Fig. 2b), all N–H bonds are split heterolytically, so the bonding electrons are assigned to the more electronegative bonding partner, nitrogen in this case. This ionic limit then leads to oxidation numbers of N^−III^ and H^+I^.

Going from classical Lewis formulae to quantum chemistry, we can quantify the number of electrons located on atoms and bonds by the population analyses already introduced above. As can easily be seen in Fig. 2c, setting the weight factor *α* to zero, splitting the bonding electrons (indicated by solid red and blue lines) is symmetric, so in case of, say, the 1s–2p_*x*/*y*_ bond, both bonding partners receive half of the 0.59 bonding electrons, 0.29 each. In sum, this separation leads to Löwdin populations and charges of −0.91 for nitrogen and +0.30 for hydrogen.

If the weighting factor *α* is chosen to be larger, the partitioning of the bonding population becomes heterolytical. As the 1s orbital of hydrogen is significantly higher in energy compared to the valence orbitals of nitrogen, the electrons are shifted strictly towards nitrogen, in line with the empirical expectation from electronegativities. For the 1s–2p_*x*/*y*_ bond, the total 0.59 bonding electrons are thus shifted completely to nitrogen, mirroring the recipe of empirical oxidation numbers. In the end, the ON^*ai*^ derived from this algorithm (H^+0.75^ and N^−2.25^) match the empirical numbers a lot closer than the respective Löwdin charges. At this point, it should be noted that fractional ON^*ai*^ are an inherent feature of the population analyses used in the calculus. While integer oxidation numbers are a consequence of the classical derivation, this does not apply to quantum chemistry. Analogously, Mulliken/Löwdin charges and populations as well as bond orders by means of ICOBI have fractional values in the vast majority of cases, especially in intermetallic phases as presented in the following.

### 3.2. Similarities and differences

**Fig. 3:**
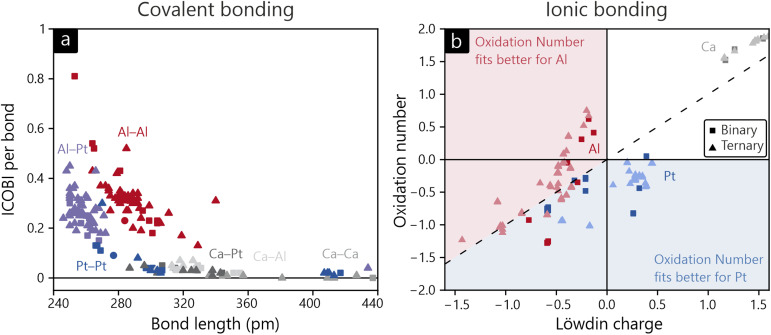
(a) Bond length plotted *versus* the ICOBI per bond. (b) *Ab initio* oxidation number plotted *versus* Löwdin charge.

Before we go into detail, we want to give an overview of all compounds included in our study, as summarized in Fig. 3. Starting with covalent bonding, Fig. 3a sets the ICOBI of each contact in relation to the respective interatomic distance. In this depiction, several trends are apparent: (1) bond orders steadily decrease with increasing interatomic distance, even though outliers are apparent. While individual elements and crystal structures affect the relationship, the overall trend is clearly visible, especially inside certain bond types. (2) Al–Al bonds are longer than Al–Pt bonds with the same bond strength, while Pt–Pt bonds are significantly weaker. Caused by the relative sizes of Al and Pt valence orbitals, this was already found for individual compounds, but the present summary shows this observation on a much wider scale. (3) Interactions involving Ca do not contain significant covalency. In the given compounds, the Ca atoms act as electron donors and can be formally identified as Ca^2+^, unable to form any covalent bonds due to its closed-shell (4s^0^) configuration.

The ionic bonding analyses in this study are conducted based on Löwdin charges and, additionally, using the newly introduced *ab initio* oxidation numbers ON^*ai*^. Their relation is shown in Fig. 3b. In all examined cases, Ca purely serves as an electron donor with charges and oxidation numbers close to the ideal +2. For Al and Pt, simple expectations are not met, however. Based on electronegativities of Al and Pt, one would assume (partially) cationic Al and anionic Pt. Fig. 3b reveals that all of the examples in our examination possess negatively charged aluminum and positively charged platinum atoms, in contrast to electronegativities of Al and Pt, respectively, 1.61 and 2.28 on Pauling's scale.^80^ Since Löwdin charges do not reflect the energetic orbital arrangement and symmetrically attribute the density of bonding electrons to both bonding partners, charges do not trivially turn into oxidation states. This disagreement is solved by introducing a weighting scheme based on atomic orbital energies (see the explanation above), ultimately leading to the formulation of *ab initio* oxidation numbers. Although these oxidation numbers are often fractional, not integer like the classical counterpart, the overall fit is significantly improved. The majority of Al atoms reside in the top left part of Fig. 3b and Pt atoms in the bottom right part. This picture can be traced back to an increased bonding electron transfer from Al to Pt that is revealed when going from Löwdin charges to oxidation numbers. As most of the systems provided in Fig. 3 follow the expected course of bond strength *vs.* bond length, we will focus the following more detailed discussion of our results on the most interesting members of the Ca–Al–Pt family. For reference, data on all compounds are summarized in the SI.

### 3.3. The elements

**Fig. 4:**
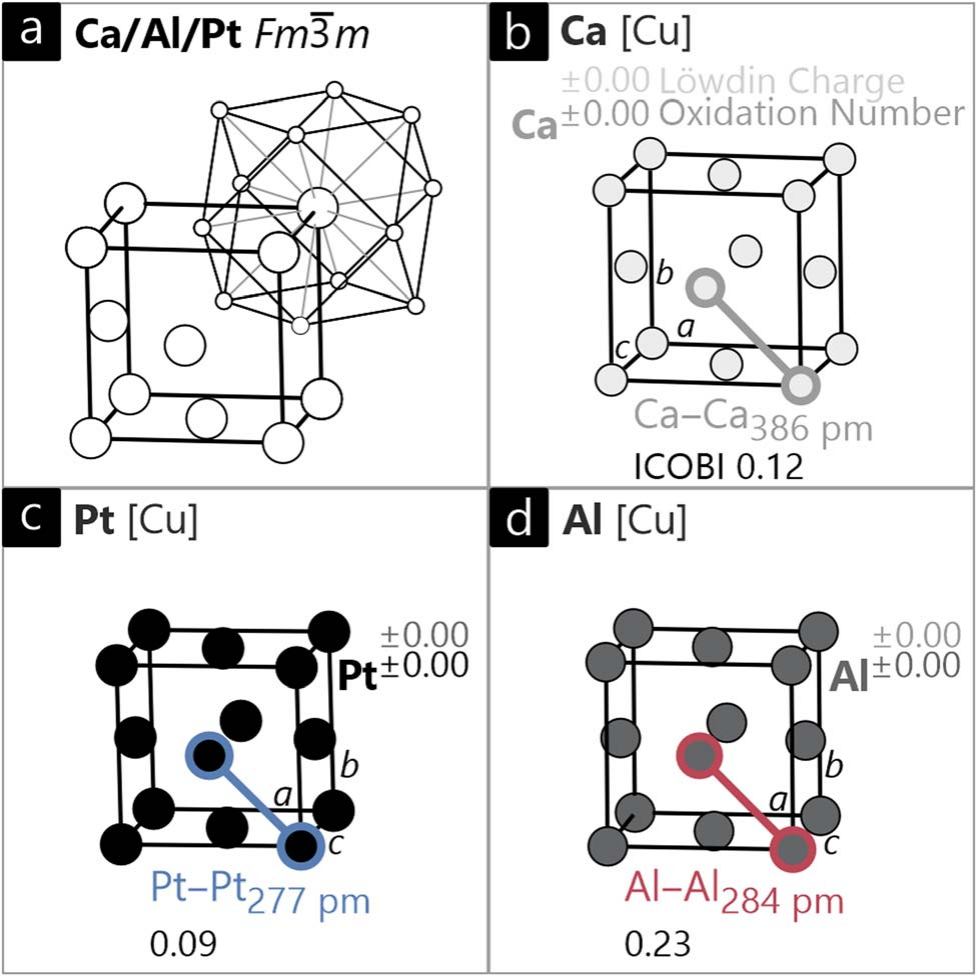
(a) Unit cell of elemental Ca, Al and Pt (Cu type, *Fm*3̄*m*) depicted with the corresponding coordination environment. Unit cells of (b) cubic Ca, (c) cubic Pt and (d) cubic Al are presented with Löwdin charges, *ab initio* oxidation numbers (ON^*ai*^), interatomic distances and respective ICOBI values.

All three elements crystallize in their stable allotropes at room temperature and ambient pressure, *i.e.*, in the cubic crystal system with the face centered space group *Fm*3̄*m*, adopting the Cu type structure (Fig. 4a).^61–63^ The DFT-optimized interatomic distances are 284 pm for Pt, 286 pm for Al, and 393 pm for Ca. In all three cases, these distances are longer compared to the sum of the covalent radii (Pt: 258 pm; Al: 250 pm; Ca: 348 pm (ref. 80)), not too surprising taking into account the larger coordination numbers.‡‡Note that we chose covalent radii here on purpose. While the metallic radii exactly match the interatomic distances by definition, we want to contextualize covalent bonding properties that are better described by covalent radii as their sum corresponds to the length of a single bond that has ICOBI = 1.

Based on the interatomic distances, one would expect rather weak covalent interactions to the neighbors, which can easily be verified using the ICOBI values of the nearest-neighbor bonds: Al–Al has the largest value of 0.23, Ca–Ca (0.12) and Pt–Pt (0.09) are smaller, indicating even weaker covalent bonding in these metals.^81^ If we sum up all of the respective bond indices, we arrive at the total bond capacity of an atom that equals its valence, similar to the empirical bond valence sum.^82^ Including only the 12 nearest-neighbor interactions, the valences are 2.76 for Al, 1.44 for Ca, and 1.08 for Pt.

This relation of rather small metal–metal bond orders and valences can easily be rationalized: if we consider elemental calcium, each Ca atom directly bonds with 12 nearest neighboring atoms, meaning that two valence electrons per Ca atom are distributed over all of these bonding partners. Consequently, the expected number of “shared” electrons between two Ca atoms will be around 2/12 ≈ 0.17, corresponding to a Ca–Ca bond order of *ca.* 0.08, as mirrored by ICOBI.

Naturally, none of these compounds show any electron transfer as all Löwdin charges are ±0. This agrees with the *ab initio* oxidation numbers (ON^*ai*^) that all have a value of ±0, a trivial consequence of an element.

### 3.4. Binary phases

As for the binary phases, stable and, where possible, multiple-times reported (according to the Pearson database^60^) representatives from each binary system were selected: CaAl_2_ (ref. 83) and CaAl_4_ (ref. 84) for the Ca–Al system, CaPt_2_ (ref. 65) for the Ca–Pt system and finally Al_2_Pt and AlPt for the Al–Pt system.

**Fig. 5:**
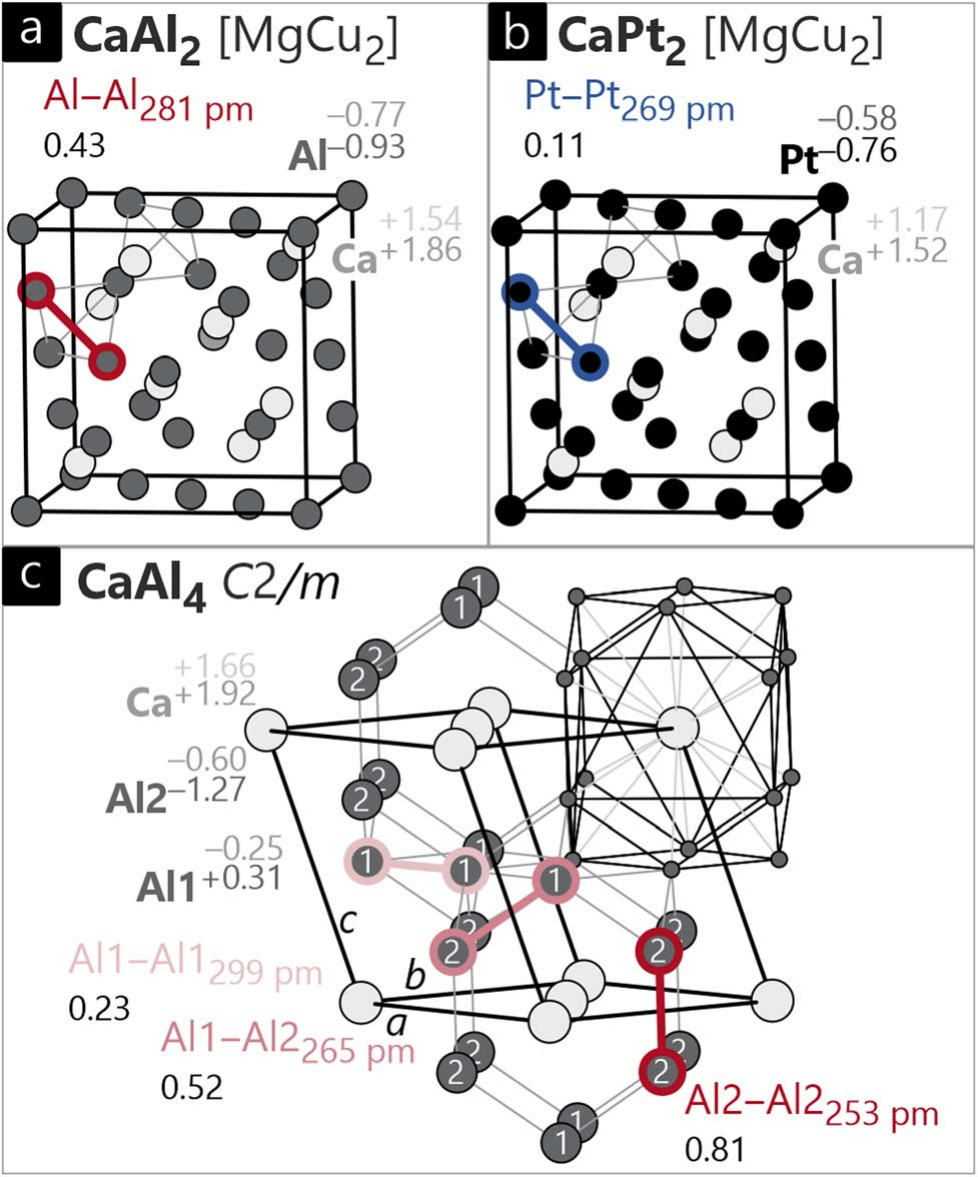
Unit cells of (a) cubic CaAl_2_ and (b) CaPt_2_, both of MgCu_2_ type (*Fd*3̄*m*), and (c) monoclinic CaAl_4_ (CaGa_4_ type, *C*2/*m*). Ca, Al and Pt atoms are shown in light grey, dark grey and black circles, respectively. The Löwdin charges, *ab initio* oxidation numbers (ON^*ai*^), interatomic distances and respective ICOBI values for the different structures are also provided.

CaAl_2_ and CaPt_2_ both adopt the cubic Laves phase (MgCu_2_ type; *Fd*3̄*m*).^85^ Here, Al_4_/Pt_4_ tetrahedra are found, which are connected *via* all four corners to a network. The Ca atoms reside in cavities of the said framework (Fig. 5a and b). The Al–Al distances in CaAl_2_ are 281 pm and therefore are well in line with elemental Al.

Despite the similar interatomic distance, the ICOBI (0.43) of this contact is larger by a factor of two, and this is directly related to the coordination number of six, as compared to CN = 12 in the fcc structure. Speaking of ionicity, one would expect the formation of aluminides, which is directly reflected in significant Löwdin charges. The Ca atoms exhibit positive charges with +1.54 for CaAl_2_, and Al anions are formed with a charge of −0.77. This charge transfer is even more pronounced when looking at the ON^*ai*^ that are +1.86 and −0.93, corresponding to Ca^+II^ and Al^−I^. A recent review on binary alkaline-earth trielides utilizing the Bader formalism observed a similar charge transfer.^86^

In isostructural CaPt_2_, the Pt–Pt distances are 270 pm and therefore are significantly shorter compared to elemental Pt, suggesting covalent bonding interaction. Indeed, the same picture is evident as found in CaAl_2_: covalently bonded Pt–Pt and rather ionic Ca–Pt interactions. Yet, there are certain differences addressed in the following: while the formation of cationic Ca and anionic Pt is given by both Löwdin charges and ON^*ai*^, the quantitative charge transfer does not match the empirical expectations. Based on electronegativity, one would assume more negative Pt compared to Al, but both the Löwdin charge and ON^*ai*^ are smaller than the respective values of Al in CaAl_2_.

From a quantum-mechanical point of view, however, elemental Al (3s^2^ 3p^1^) possesses a less than half-filled valence shell, while the valence of Pt (6s^2^ 5d^8^) is more than half-filled. Following the (generalized) octet rule and correlation arguments, adding an electron to Pt is energetically less favorable than adding an electron to Al. This destabilization is also visible in the (I)COBI of the Pt–Pt contacts. Comparing elemental Pt and CaPt_2_, the ICOBI is larger for the shorter contact in the binary compound, but only by a small amount – especially in comparison with Al/CaAl_2_. Pushing electrons from Ca onto Pt leads to the population of antibonding levels in the Pt–Pt interactions, thereby weakening the individual bonds.

CaAl_4_ adopts a monoclinic structure (CaGa_4_ type, *C*2/*m*) that can be derived from the tetragonal BaAl_4_ type structure by a group-subgroup formalism.^64^ Once again, the Al atoms form a network with the Ca atoms residing in cavities (Fig. 5c). Here, one would naively consider the shorter Al–Al contacts to be covalent bonding interactions, while the longer ones would be considered non-bonding. Interestingly, the rather long Al1–Al1 contacts still exhibit significant ICOBI values of about 0.23. The Al1–Al2 distances are significantly shorter, leading to an increase in the ICOBI values to slightly above the half bond order. The shortest distance, finally, is the Al2–Al2 interaction between the layered fragments with a stunningly large ICOBI value of 0.81, indicating a very strong covalent bond, approaching the classical single bond order. The Löwdin charges finally reflect the coordination environments: while Al1 exhibits in a wider sense a coordination number of 12 (Al1@Al2_4_Al1_4_Ca_4_), Al2 has a coordination number of 9 (Al2@Al2Al1_4_Ca_4_) with overall shorter distances and therefore a higher overall Löwdin charge. This trend has also been observed by charge transfer analyses based on the definition by Bader.^64,86^ Interestingly, the ON^*ai*^ further differentiate between both Al sites resulting in an anionic Al2 (−1.27) and a cationic Al1 (+0.31), solid-state disproportionation, so to speak. In order to understand this behavior, a fundamental solid-state periodic property needs to be recalled, namely the Madelung field. Pure electrostatics leads to an attractive (=stabilizing) force between cations and anions. Considering the rather short Al2–Ca distance relative to Al1–Ca, it is safe to assume a stronger stabilization of Al2, in turn lowering the orbital energies of Al2, anion-like. Thus, the bonding electrons of the Al1–Al2 bonds move to the more electronegative Al2 atoms, and this ultimately leads to the formation of Al1 cations and Al2 anions, at least formally.

**Fig. 6:**
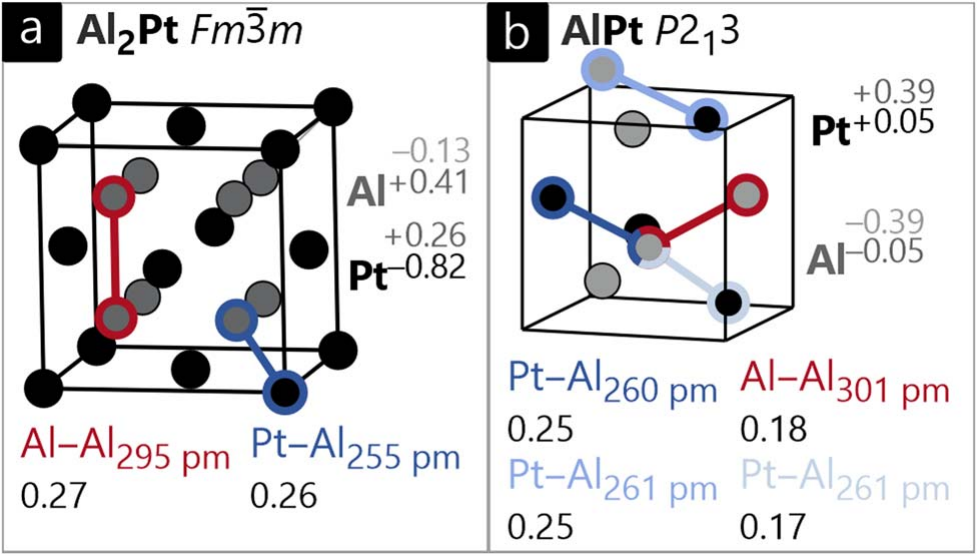
Unit cells of (a) cubic Al_2_Pt (CaF_2_ type, *Fm*3̄*m*), and (b) cubic AlPt (FeSi type, *P*2_1_3). Al and Pt atoms are shown in dark grey and black circles, respectively. The Löwdin charges, *ab initio* oxidation numbers (ON^*ai*^), interatomic distances and respective ICOBI values for the different structures are also provided.

Finally, the compounds from the Al–Pt system should be addressed (Fig. 6). When looking at the bond indices in Al_2_Pt (Fig. 6a), the Al–Pt contacts are significantly shorter compared to the Al–Al interactions, even though both exhibit similar ICOBI values of 0.27 and 0.26. The ionic nature of this compound, as given by the Löwdin charges, does not reflect the empirical expectation from electronegativities. This tentative disagreement can immediately be solved by the respective oxidation numbers ON^*ai*^. Using orbital energies as criteria and heterolytic charge allocation, bonding electrons of the Al–Pt bonds are transferred from Al 3p to the lower-lying Pt 5d orbitals. This way, the positively charged Pt (+0.26) adopts an anionic oxidation number (−0.82) and the negatively charged Al (−0.13) has a cationic oxidation number (+0.41). Such a charge transfer of 0.54 electrons per Al atom to Pt is only revealed by the combination of an atom-centered (ionic) and a bond-centered (covalent) analysis, as explained above.

In AlPt (Fig. 6b), a contrasting picture is observed. There are three distinct Al–Pt contacts, suggesting similar bonding based on the distances. The first two interactions indeed have identical ICOBI values of 0.25, and the third interaction, which is almost identical in length, only has an ICOBI value of 0.17. This puzzling difference shall be investigated using the energy-dependent COBI, as shown in Fig. S2. At a quick glance, we identify antibonding levels directly below the Fermi level, and their amount is largest in the weakest Al–Pt bond, so the small ICOBI is not due to decreased orbital interaction but a shift from occupied bonding to antibonding levels. Note that this destabilizing part is counteracted by the remaining Al–Pt bonds that have a multiplicity of three in contrast to the “weakest” bond appearing only once per polyhedron. On a descriptive level, we may infer that the remaining contacts are stabilized at the cost of the weak bond resulting in a net stabilizing effect. Ionicity, on the other hand, is more transparent. As also found for Al_2_Pt above, the Löwdin charges show counter-intuitive Al anions and Pt cations. This discrepancy in terms of electronegativities is again resolved by the oxidation numbers being significantly smaller than the Löwdin charges. This time, however, the oxidation numbers are almost neutral with ±0.05, and they do not mirror distinct ions.

In general, it can be observed that the charge transfer from Al to Pt increases with increasing Al content. This is in line with recent studies on the Al–Pt system based on QTAIM,^87,88^ although the absolute values reported in literature are larger than our Löwdin charges and ON^*ai*^.

### 3.5. Ternary phases

In the following paragraphs, the crystal structures of some of the compounds listed in Table S5 will be discussed exemplarily alongside similarities, differences and specific features that will be highlighted by the bonding analysis later.

**Fig. 7:**
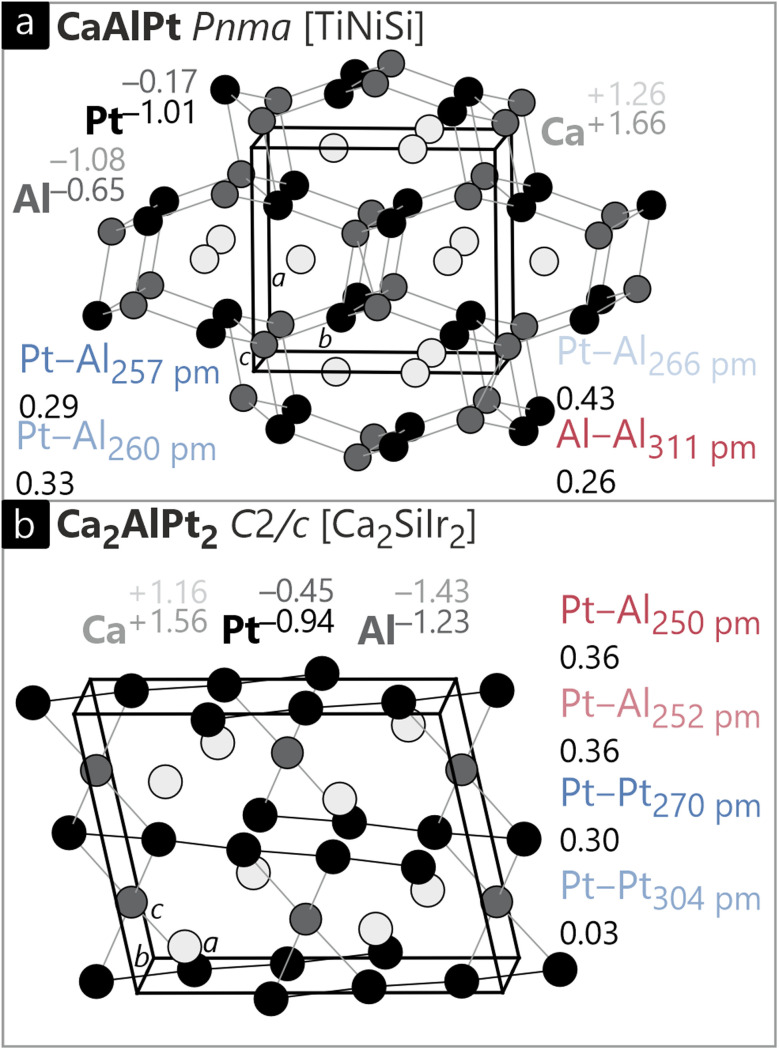
Unit cells of (a) orthorhombic CaAlPt (TiNiSi type, *Pnma*) and (b) monoclinic Ca_2_AlPt_2_ (Ca_2_SiIr_2_ type, *C*2/*c*) with corresponding Löwdin charges, *ab initio* oxidation numbers (ON^*ai*^), and ICOBI values for selected bonds. Ca, Al and Pt atoms are shown in light grey, white and black circles, respectively.

We start with equiatomic CaAlPt^89,90^ which crystallizes in the orthorhombic crystal system with the space group *Pnma* and adopts the TiNiSi type structure. The crystal structure can be described based on a network formed by the Al and Pt atoms with the Ca atoms residing in cavities (Fig. 7a). The Al–Pt distances are relatively short within the network; additionally, elongated Al–Al distances can be observed; however, no Pt–Pt interactions are present. When compared with the distance discussions above, at least Al–Pt bonds are present. Since the Al to Pt ratio is 1 : 1, forming Al–Pt interactions is a necessity, while Al–Al and Pt–Pt interactions are not necessarily required. The Ca–Al and Ca–Pt distances are relatively short, but clearly distinct to at least the Al–Pt contacts and in the range of the sum of covalent radii (*vide infra*). Based on the different distances, one can interpret this structure as a polyanionic [AlPt]^*δ*−^ network with Ca^*δ*+^ cations residing in the cavities. This is a frequently observed picture, especially in the Al-rich compounds.

The Löwdin charges underline the picture of a polyanionic network (Ca: +1.26; Al: −1.08; Pt: −0.17); however, as already observed for the binary compounds in the Al–Pt system, *i.e.*, the Al atoms carry a higher negative charge counterintuitive to the electronegativities. In this case, both the Al and the Pt atoms are negatively charged and also show an anionic oxidation number. The ON^*ai*^, however, are in better agreement with the trend derived from the electronegativities and arrive at −1.01 for Pt while Al yields −0.65. The interatomic Al–Pt distances are in a similar range as the ones observed in the binaries, but overall higher ICOBI values are observed. This can be attributed to the additional electron transfer from the Ca atoms onto the polyanion, filling bonding levels. Interestingly, the longest Al–Pt contact exhibits the strongest covalency (0.43). Besides the Al–Pt contacts also one relatively long Al–Al distance is present. This interaction would not be considered bonding with respect to the distances found in elemental Al or the sum of the covalent radii, but the ICOBI value of 0.26 clearly indicates bonding interactions.

We now draw our attention to monoclinic Ca_2_AlPt_2_ (Fig. 7b, Ca_2_SiIr_2_ type). Here, there are chains of Pt atoms with alternating shorter and longer distances. These chains run parallel to the *ab* plane with an angle of 63.6° between them. The Al atoms connect two of the chains with rather short Al–Pt contacts, always bridging the longer Pt–Pt distances. The Ca atoms reside in the created cavities. This compound was reported by Doverbratt and coworkers who also analyzed the bonding.^91^ The authors highlight the linear platinum chains that distort pairwise into shorter dumbbells and longer Pt⋯Pt contacts. When looking at the ICOBI values (Fig. 7b), 0.30 can be found for the short Pt–Pt distance, while only 0.03 is calculated for the long distance. This is in line with Doverbratt *et al.*;^91^ however, their bond orders were 0.48 and 0.26 according to the non-quantum-chemical bond-length bond-strength approach by Brese and O'Keeffe.^82^ They state that “the interactions within the anionic substructures are essentially nonbonding (or slightly bonding) and indicate that the electrostatic repulsions are suppressed when replacing the anionic Ge bridging elements by cationic Al atoms”. This can be clearly seen by the ICOBI values of 0.36 for the Al–Pt interactions. The ionic bonds in Ca_2_AlPt_2_ suggest a similar decomposition as found in the previously discussed CaAlPt. Ca is cationic, and Al and Pt form an anionic network with Al being slightly more negatively charged than Pt. As found before, the oxidation numbers reveal a polarization of the Al–Pt bonds that results in more negative Pt and less negative Al.

**Fig. 8:**
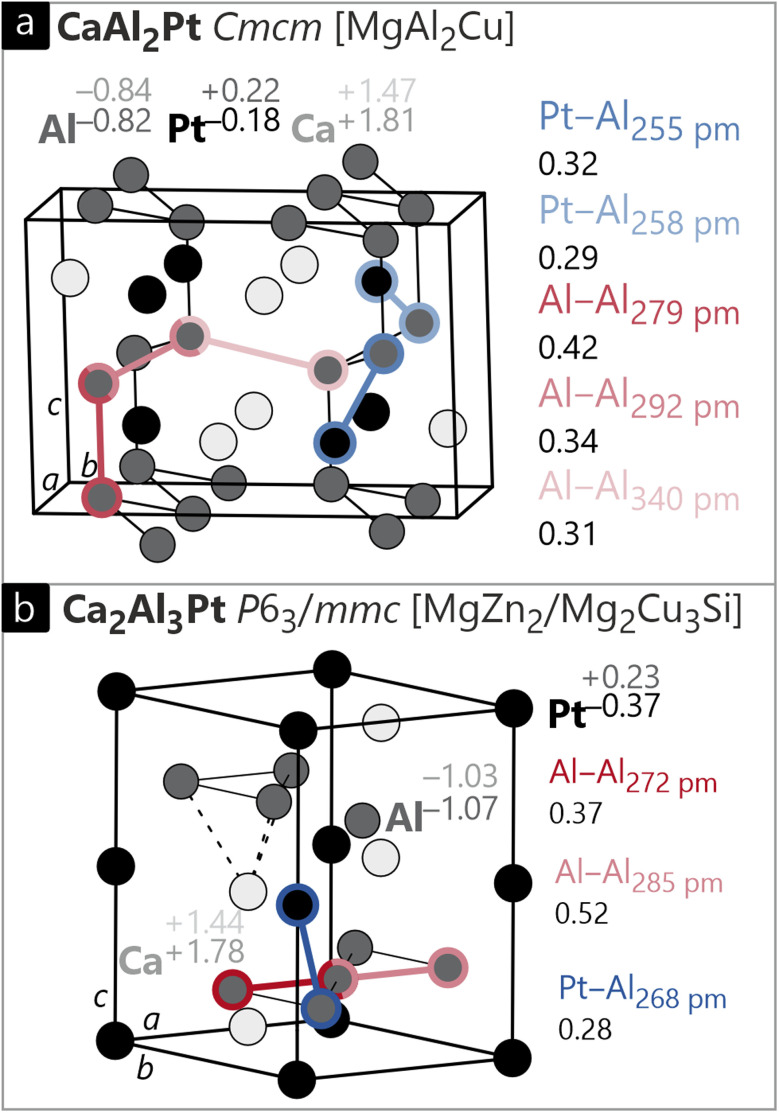
Unit cell of (a) orthorhombic CaAl_2_Pt (MgCuAl_2_ type, *Cmcm*) and (b) hexagonal Ca_2_Al_3_Pt (MgZn_2_/Mg_2_Cu_3_Si type, P6_3_/mmc). The Löwdin charges, *ab initio* oxidation numbers (ON^*ai*^), interatomic distances and corresponding ICOBI values for different bonds are indicated. Ca, Al and Pt atoms are shown in light grey, dark grey and black circles, respectively.

When finally going to CaAl_2_Pt (orthorhombic, MgAl_2_Cu type, *Cmcm*, Fig. 8a),^92^ a compound with Al being the majority element is discussed, so can distinct Al–Al bonding be observed? When only focusing on the arrangement of the Al atoms, we see corrugated honeycomb layers all in boat conformation with the Pt atoms residing in the center of each hexagon. As described before, the Al–Pt contacts are the shortest ones observed in the structure, while the Al–Al distances are slightly longer. This generates [Al_2_Pt] layers which are separated by the Ca atoms leading to Ca–Pt distances of 314 pm and Ca–Al distances of 323 and 342 pm. The shortest distance between the layers not involving Ca atoms is Al–Al = 349 pm. From a crystal-chemical point of view, one would discard this distance as not being involved in any bonding. Interestingly, the same distance elongates to 415 pm in SrAl_2_Pt^92^ and even further to 471 pm in BaAl_2_Pt.^92^ While CaAl_2_Pt is quite stable towards moisture, SrAl_2_Pt and BaAl_2_Pt decompose rapidly, so does the short Al–Al distance stabilize CaAl_2_Pt? In the rare-earth representatives, this contact decreases down to 281 pm for ScAl_2_Pt,^93^ which is well in line with what is considered a bonding interaction based on structural considerations. In the relaxed DFT model (Fig. 8a), the distance in CaAl_2_Pt is slightly shorter (340 pm); however, the ICOBI value is 0.31, that is about a third of a single bond. This value is especially remarkable since the shorter Al–Al contact with a length of 292 pm has a similar ICOBI of 0.34 despite the significant bond-length difference, which addresses a considerable covalency to the longer interaction. Talking of ionicity, the Löwdin charges suggest an anionic Al–Pt network that contains Ca cations. The positive sign of the Pt charge (+0.22) turns into an anionic ON^*ai*^ (−0.18) that is primarily caused by a shift of bonding electrons from the Ca–Pt bonds to Pt. Al has the same values for the Löwdin charge and oxidation number.

Another interesting Al–Al interaction can be observed in Ca_2_Al_3_Pt (Fig. 8b). Here, the Al atoms form a 6^3^ Kagome net with the Pt atoms connecting two layers. Within the Al layer, there are two different interactions, the shorter one being 272 and the longer being 285 pm. Puzzling, the shorter interaction exhibits the smaller ICOBI value of 0.37, while the significantly longer distance shows a significantly higher covalency, with 0.52 being the highest ICOBI value for an Al–Al interaction observed. The lower ICOBI for the shorter bond can be traced back to (occupied) antibonding contribution, below the Fermi energy. Note that a shorter bond length may increase orbital overlap, but this also holds for antibonding interactions that decrease the bond strength as in the present example. Why, however, does this unexpected course of bond strength *vs.* bond length appear in the first place for a compound with only one crystallographic site per element? To answer, we take a quick look at the crystal structure: the triangles formed by the longer Al–Al bonds are capped by a calcium atom (dashed lines in Fig. 8b), essentially forming a trigonal pyramid; this structural feature does not exist for the shorter Al–Al contacts. It is safe to assume that the cationic presence of Ca^2+^ stabilizes the bonds in the anionic Al-network, thus leading to an increased ICOBI despite the longer Al–Al distance. Charges and ON^*ai*^ of Ca_2_Al_3_Pt match our findings from the previous discussion.

## 4. Conclusions

In this work, we presented a thorough investigation of the bonding properties for members of the Ca–Al–Pt family starting with the elements, then continuing *via* binary to ternary compounds. As already found in previous work, the chemical bonding in intermetallic phases is not necessarily analogous to simple molecular compounds due to complexities of the solid state and metallicity. Using the toolbox provided by LOBSTER, we investigated covalent bonding by the crystal orbital bond index and ionic bonding by means of Löwdin charges and *ab initio* oxidation numbers. By comparison of both descriptors for ionicity, we demonstrated the strength of the oxidation numbers that directly relate to oxidation states covered in any basic chemistry course. The ability to calculate such numbers promises unexpected insights into intermetallic phases where classical concepts do not apply.

## Author contributions

All authors have accepted responsibility for the entire content of this submitted manuscript and approved the submission.

## Conflicts of interest

The authors declare no conflicts of interest regarding this article.

## Supplementary Material

SC-OLF-D5SC02993G-s001

## Data Availability

Crystallographic data can be found in the primary articles cited throughout the manuscript, further information is given in the SI. If any data is missing, it can be requested from the corresponding authors upon reasonable request. See DOI: https://doi.org/10.1039/d5sc02993g.

## References

[cit1] Altenpohl D. , Aluminium und Aluminiumlegierungen, Springer, Berlin, Heidelberg, Germany, 1965

[cit2] Kammer C. , Aluminium-Taschenbuch 1: Grundlagen und Werkstoffe, Aluminium-Zentrale: Düsseldorf, Germany, 1995

[cit3] Ostermann F. , Anwendungstechnologie Aluminium. Springer Vieweg: Berlin, Heidelberg, Germany, 2014

[cit4] Janka O. , Metallic light-weight alloys: Al, Ti, Mg, in Applied Inorganic Chemistry, ed. Pöttgen, R., Jüstel, T. and Strassert, C., De Gruyter, Berlin, Germany, 2022, pp. 158–173

[cit5] Buchner M. and Janka O., Volume 1 From Construction Materials to Technical Gases – Chapter 2.7 Be and Be alloys, in Applied Inorganic Chemistry, ed. Pöttgen, R., Jüstel, T. and Strassert, C. A., De Gruyter, Berlin, 2023, pp. 221–228

[cit6] Engel S. and Janka O., Volume 1 From Construction Materials to Technical Gases - Chapter 2.10 Shape memory alloys, in Applied Inorganic Chemistry, ed. Pöttgen, R., Jüstel, T. and Strassert, C. A., De Gruyter, 2023, pp. 255–264

[cit7] Engel S. and Janka O., Volume 1 From Construction Materials to Technical Gases - Chapter 2.11 Bulk metallic glasses, in Applied Inorganic Chemistry, ed. Pöttgen, R., Jüstel, T. and Strassert, C. A., De Gruyter, 2023, pp. 265–270

[cit8] Strnat K., Hoffer G., Olson J., Ostertag W., Becker J. J. (1967). A Family of New Cobalt-Base Permanent Magnet Materials. J. Appl. Phys..

[cit9] Croat J. J., Herbst J. F., Lee R. W., Pinkerton F. E. (1984). High-energy product Nd-Fe-B permanent magnets. Appl. Phys. Lett..

[cit10] Rahmel A., Schütze M., Quadakkers W. J. (1995). Fundamentals of TiAl oxidation – A critical review. Mater. Corros..

[cit11] Kresse G., Schmid M., Napetschnig E., Shishkin M., Köhler L., Varga P. (2005). Structure of the Ultrathin Aluminum Oxide Film on NiAl(110). Science.

[cit12] Datta P. K. , Du H. L., Burnell-Gray J. S. and Ricker R. E., Corrosion of Intermetallics, in Corrosion: Materials, ASM International, Materials Park, OH, USA, 2005, vol. 13B

[cit13] Jozwik P., Polkowski W., Bojar Z. (2015). Applications of Ni_3_Al Based Intermetallic Alloys – Current Stage and Potential Perceptivities. Materials.

[cit14] Armbrüster M., Kovnir K., Behrens M., Teschner D., Grin Y., Schlögl R. (2010). Pd–Ga Intermetallic Compounds as Highly Selective Semihydrogenation Catalysts. J. Am. Chem. Soc..

[cit15] Armbrüster M., Kovnir K., Friedrich M., Teschner D., Wowsnick G., Hahne M., Gille P., Szentmiklósi L., Feuerbacher M., Heggen M., Girgsdies F., Rosenthal D., Schlögl R., Grin Y. (2012). Al_13_Fe_4_ as a low-cost alternative for palladium in heterogeneous hydrogenation. Nat. Mater..

[cit16] Antonyshyn I., Sichevych O., Rasim K., Ormeci A., Burkhardt U., Titlbach S., Schunk S. A., Armbrüster M., Grin Y. (2018). Chemical behaviour of CaAg_2_ under ethylene epoxidation conditions. Eur. J. Inorg. Chem..

[cit17] Hodge K. L., Goldberger J. E. (2019). Transition Metal-Free Alkyne Hydrogenation Catalysis with BaGa_2_, a Hydrogen Absorbing Layered Zintl Phase. J. Am. Chem. Soc..

[cit18] Li J., Sun S. (2019). Intermetallic Nanoparticles: Synthetic Control and Their Enhanced Electrocatalysis. Acc. Chem. Res..

[cit19] Armbrüster M. (2020). Intermetallic compounds in catalysis – a versatile class of materials meets interesting
challenges. Sci. Technol. Adv. Mater..

[cit20] Kanatzidis M. G., Pöttgen R., Jeitschko W. (2005). The metal flux: A preparative tool for the exploration of intermetallic compounds. Angew. Chem., Int. Ed..

[cit21] Steurer W. and Dshemuchadse J., Intermetallics. Union of Crystallography, Oxford University Press, Oxford, 2016

[cit22] Pöttgen R. and Johrendt D., Intermetallics - Synthesis, Structure, Function, De Gruyter, Berlin, Boston, 2019

[cit23] Oganov A. R. , Modern Methods of Crystal Structure Prediction, Wiley-VCH, Weinheim, 2010

[cit24] George J., Hautier G. (2021). Chemist versus Machine: Traditional Knowledge versus Machine Learning Techniques. Trends Chem..

[cit25] Dahliah D., Brunin G., George J., Ha V.-A., Rignanese G.-M., Hautier G. (2021). High-throughput computational search for high carrier lifetime, defect-tolerant solar absorbers. Energy Environ. Sci..

[cit26] George J., Petretto G., Naik A., Esters M., Jackson A. J., Nelson R., Dronskowski R., Rignanese G.-M., Hautier G. (2022). Automated Bonding Analysis with Crystal Orbital Hamilton Populations. ChemPlusChem.

[cit27] Ganose A. M., Sahasrabuddhe H., Asta M., Beck K., Biswas T., Bonkowski A., Bustamante J., Chen X., Chiang Y., Chrzan D. C., Clary J., Cohen O. A., Ertural C., Gallant M. C., George J., Gerits S., Goodall R. E. A., Guha R. D., Hautier G., Horton M., Inizan T. J., Kaplan A. D., Kingsbury R. S., Kuner M. C., Li B., Linn X., McDermott M. J., Mohanakrishnan R. S., Naik A. N., Neaton J. B., Parmar S. M., Persson K. A., Petretto G., Purcell T. A. R., Ricci F., Rich B., Riebesell J., Rignanese G.-M., Rosen A. S., Scheffler M., Schmidt J., Shen J.-X., Sobolev A., Sundararaman R., Tezak C., Trinquet V., Varley J. B., Vigil-Fowler D., Wang D., Waroquiers D., Wen M., Yang H., Zheng H., Zheng J., Zhu Z., Jain A. (2025). Atomate2: modular workflows for materials science. Digital Discovery.

[cit28] Grin Y. , Crystal Structure and Bonding in Intermetallic Compounds, in Comprehensive Inorganic Chemistry II, ed. Reedijk, J. and Poeppelmeier, K., Elsevier, Amsterdam, 2nd edn, 2013, pp. 359–373

[cit29] Burdett J. K. (1988). Perspectives in structural chemistry. Chem. Rev..

[cit30] Nesper R. (1991). Chemische Bindungen - intermetallische Verbindungen. Angew. Chem..

[cit31] Nesper R. (1991). Bonding Patterns in Intermetallic Compounds. Angew. Chem., Int. Ed..

[cit32] Burdett J. K. , Chemical Bonds: A Dialog, John Wiley & Sons Ltd, Chichester, England, 1997

[cit33] Albright T. A. , Burdett J. K. and Whangbo M.-H., Orbital Interactions in Chemistry, Wiley & Sons Ltd, Hoboken, New Jersey, 2013

[cit34] Miller G. J. , Zhang Y. and Wagner F. R., Chemical Bonding in Solids, in Handbook of Solid State Chemistry, Wiley-VCH, Weinheim, 2017, pp. 405–489

[cit35] Smetana V., Rhodehouse M., Meyer G., Mudring A.-V. (2017). Gold Polar Intermetallics: Structural Versatility through Exclusive Bonding Motifs. Acc. Chem. Res..

[cit36] Lin Q., Miller G. J. (2018). Electron-poor polar intermetallics: Complex structures, novel clusters, and intriguing bonding with pronounced electron delocalization. Acc. Chem. Res..

[cit37] Jones R. O. (2022). The chemical bond in solids – revisited. J. Phys.: Condens. Matter.

[cit38] Steinberg S. (2025). Tellurides as Zintl phases?. J. Phys.: Condens. Matter.

[cit39] Karpov A., Nuss J., Wedig U., Jansen M. (2003). Cs2Pt: A Platinide(-II) Exhibiting Complete Charge Separation. Angew. Chem., Int. Ed..

[cit40] Jansen M. (2005). Effects of relativistic motion of electrons on the chemistry of gold and platinum. Solid State Sci..

[cit41] Peer W. J., Lagowski J. J. (1978). Metal-ammonia solutions. 11. Au^–^, a solvated transition metal anion. J. Am. Chem. Soc..

[cit42] Jansen M. (2008). The chemistry of gold as an anion. Chem. Soc. Rev..

[cit43] Laves F. (1930). XVI. Die Bau-Zusammenhänge innerhalb der Kristallstrukturen. Z. Kristallogr..

[cit44] Pauling L. (1929). The principles determining the structure of complex ionic crystals. J. Am. Chem. Soc..

[cit45] Pauling L. (1947). Atomic Radii and interatomic distances in metals. J. Am. Chem. Soc..

[cit46] Pauling L. , The nature of the chemical bond and the structure of molecules and crystals: an introduction to modern structural chemistry, Cornell University Press, Ithaca, NY, USA, 1960

[cit47] Hume-Rothery W. , The metallic state: electrical properties and theories. Clarendon Press, Oxford, Great Britai, 1931

[cit48] Dronskowski R. , Chemical Bonding, Walter De Gruyter, Berlin/Boston, 2023

[cit49] Hughbanks T., Hoffmann R. (1983). Chains of trans-edge-sharing molybdenum octahedra: metal-metal bonding in extended systems. J. Am. Chem. Soc..

[cit50] Dronskowski R., Blöchl P. E. (1993). Crystal orbital Hamilton populations (COHP): energy-resolved visualization of chemical bonding in solids based on density-functional calculations. J. Phys. Chem..

[cit51] Nelson R. , Ertural C., Müller P. C. and Dronskowski R., Chemical bonding with plane waves, in Comprehensive Inorganic Chemistry III, ed. Reedijk, J. and Poeppelmeier, K. R., Elsevier, Oxford, 3rd edn, 2023, pp. 141–201

[cit52] Bader R. F. W. (1991). A quantum theory of molecular structure and its applications. Chem. Rev..

[cit53] Savin A., Nesper R., Wengert S., Fässler T. F. (1997). ELF: The Electron Localization Function. Angew. Chem., Int. Ed..

[cit54] Fredrickson D. C. (2012). DFT-chemical pressure analysis: Visualizing the role of atomic size in shaping the structures of inorganic materials. J. Am. Chem. Soc..

[cit55] Frenking G. and Shaik S., The chemical bond: fundamental aspects of chemical bonding, John Wiley & Sons, Weinheim, Germany, 2014

[cit56] Wagner F. R. and Grin Y., Chemical bonding analysis in position space, in Comprehensive Inorganic Chemistry III, ed. Reedijk, J. and Poeppelmeier, K. R., Elsevier, Oxford, 3rd edn, 2023, pp. 222–237

[cit57] Nelson R., Ertural C., George J., Deringer V. L., Hautier G., Dronskowski R. (2020). LOBSTER: Local orbital projections, atomic charges, and chemical-bonding analysis from projector-augmented-wave-based density-functional theory. J. Comput. Chem..

[cit58] Ertural C., Steinberg S., Dronskowski R. (2019). Development of a robust tool to extract Mulliken and Löwdin charges from plane waves and its application to solid-state materials. RSC Adv..

[cit59] Müller P. C., Ertural C., Hempelmann J., Dronskowski R. (2021). Crystal Orbital Bond Index: Covalent Bond Orders in Solids. J. Phys. Chem. C.

[cit60] Villars P. and Cenzual K., Pearson's Crystal Data: Crystal Structure Database for Inorganic Compounds, release 2024/25, ASM International®, Materials Park, Ohio, USA, 2024

[cit61] Hull A. W. (1917). A new method of X-ray crystal analysis. Phys. Rev..

[cit62] Hull A. W. (1919). The positions of atoms in metals. Trans. Am. Inst. Electr. Eng..

[cit63] Hull A. W. (1920). The Arrangement of Atoms in Some Common Metals. Science.

[cit64] Engel S., Gießelmann E. C. J., Schank L. E., Heymann G., Brix K., Kautenburger R., Beck H. P., Janka O. (2023). Theoretical and ^27^Al NMR spectroscopic investigations of binary intermetallic alkaline-earth aluminides. Inorg. Chem..

[cit65] Wood E. A., Compton V. B. (1958). Laves-phase compounds of alkaline earths and noble metals. Acta Crystallogr..

[cit66] Zintl E., Harder A., Haucke W. (1937). Legierungsphasen mit Fluoritstruktur (22. Mitteilung über Metalle und Legierungen). Z. Phys. Chem..

[cit67] Schubert K., Burkhardt W., Esslinger P., Günzel E., Meissner H. G., Schütt W., Wegst J., Wilkens M. (1956). Einige strukturelle Ergebnisse an metallischen Phasen. Naturwissenschaften.

[cit68] Blöchl P. E. (1994). Projector augmented-wave method. Phys. Rev. B: Condens. Matter Mater. Phys..

[cit69] Kresse G., Hafner J. (1993). Ab initio molecular dynamics for liquid metals. Phys. Rev. B: Condens. Matter Mater. Phys..

[cit70] Kresse G., Furthmüller J. (1996). Efficient iterative schemes for ab initio total-energy calculations using a plane-wave basis set. Phys. Rev. B: Condens. Matter Mater. Phys..

[cit71] Kresse G., Furthmüller J. (1996). Efficiency of ab-initio total energy calculations for metals and semiconductors using a plane-wave basis set. Comput. Mater. Sci..

[cit72] Kresse G., Joubert D. (1999). From ultrasoft pseudopotentials to the projector augmented-wave method. Phys. Rev. B: Condens. Matter Mater. Phys..

[cit73] Kresse G. , Marsman M. and Furthmüller J., Vienna Ab-initio Simulation Package VASP: the Guide, Computational Materials Physics, Faculty of Physics, Universität Wien, Vienna, Austria, 2014

[cit74] Perdew J. P., Burke K., Ernzerhof M. (1996). Generalized Gradient Approximation made simple. Phys. Rev. Lett..

[cit75] Deringer V. L., Tchougréeff A. L., Dronskowski R. (2011). Crystal Orbital Hamilton Population (COHP) Analysis As Projected from Plane-Wave Basis Sets. J. Phys. Chem. A.

[cit76] Maintz S., Deringer V. L., Tchougréeff A. L., Dronskowski R. (2013). Analytic projection from plane-wave and PAW wavefunctions and application to chemical-bonding analysis in solids. J. Comput. Chem..

[cit77] Maintz S., Deringer V. L., Tchougréeff A. L., Dronskowski R. (2016). LOBSTER: A tool to extract chemical bonding from plane-wave based DFT. J. Comput. Chem..

[cit78] Wiberg K. B. (1968). Application of the pople-santry-segal CNDO method to the cyclopropylcarbinyl and cyclobutyl cation and to bicyclobutane. Tetrahedron.

[cit79] Mayer I. (1983). Charge, bond order and valence in the AB initio SCF theory. Chem. Phys. Lett..

[cit80] Emsley J. , The Elements, Clarendon Press, Oxford University Press, Oxford, New York, 1998

[cit81] Reitz L. S., Hempelmann J., Müller P. C., Dronskowski R., Steinberg S. (2024). Bonding Analyses in the Broad
Realm of Intermetallics: Understanding the Role of Chemical Bonding in the Design of Novel Materials. Chem. Mater..

[cit82] Brese N. E., O’Keeffe M. (1991). Bond-Valence Parameters for Solids. Acta Crystallogr..

[cit83] Nowotny H., Wormnes E., Mohrnheim A. (1940). Untersuchungen in den Systemen Aluminium-Kalzium, Magnesium-Kalzium und Magnesium-Zirkon. Z. Metallkd..

[cit84] Miller G. J., Li F., Franzen H. F. (1993). The structural phase transition in calcium-aluminum compound (CaAl_4_): a concerted application of Landau theory and energy band theory. J. Am. Chem. Soc..

[cit85] Gießelmann E. C. J., Pöttgen R., Janka O. (2023). Laves phases: superstructures induced by coloring and distortions. Z. Anorg. Allg. Chem..

[cit86] Beck H. P. (2024). A review on binary alkaline-earth trielides: an cverview of compositions and structures, of their stabilities and energetics and of criteria for the validity of the Zintl-concept. Z. Anorg. Allg. Chem..

[cit87] Baranov A., Kohout M., Wagner F. R., Grin Y., Bronger W. (2007). Spatial chemistry of the aluminium–platinum compounds: a quantum chemical approach. Z. Kristallogr..

[cit88] Antonyshyn I., Sichevych O., Burkhardt U., Barrios Jiménez A. M., Melendez-Sans A., Liao Y.-F., Tsuei K.-D., Kasinathan D., Takegami D., Ormeci A. (2023). Al–Pt intermetallic compounds: HAXPES study. Phys. Chem. Chem. Phys..

[cit89] Kenfack Tsobnang P., Fotio D., Ponou S., Fon Abi C. (2011). Calcium platinum aluminium, CaPtAl. Acta Crystallogr..

[cit90] Hulliger F. (1993). On new ternary aluminides *Ln*PdAl and *Ln*PtAl. J. Alloys Compd..

[cit91] Doverbratt I., Ponou S., Zhang Y., Lidin S., Miller G. J. (2015). Linear Metal Chains in Ca_2_*M*_2_*X* (*M* = Pd, Pt; *X* = Al, Ge): Origin of the Pairwise Distortion and Its Role in the Structure Stability. Chem. Mater..

[cit92] Stegemann F., Block T., Klenner S., Zhang Y., Fokwa B. P. T., Timmer A., Mönig H., Doerenkamp C., Eckert H., Janka O. (2019). From 3D to 2D: structural, spectroscopical and theoretical investigations of the dimensionality reduction in the [PtAl_2_]^*δ*–^ polyanions of the isotypic *M*PtAl_2_ series (*M* = Ca–Ba, Eu). Chem.–Eur. J..

[cit93] Radzieowski M., Stegemann F., Doerenkamp C., Matar S. F., Eckert H., Dosche C., Wittstock G., Janka O. (2019). Correlations of crystal and electronic structure via NMR and XPS spectroscopy in the *RETM*Al_2_ (*RE* = Sc, Y, La–Nd, Sm, Gd–Tm, Lu; *TM* = Ni, Pd, Pt) series. Inorg. Chem..

