# Supplementary material for: Differences and similarities in the chemical bonding of intermetallic phases in the Ca–Al–Pt system
Source: Chem Sci. 2025 Aug 28;16(38):17900–10. doi: 10.1039/d5sc02993g (PMC12415627; doi:10.1039/d5sc02993g)
Supplement: SC-016-D5SC02993G-s001 [file SC-016-D5SC02993G-s001.pdf]

Supplementary Information (SI) for Chemical Science.
This journal is © The Royal Society of Chemistry 2025

Differences and similarities in the chemical bonding of intermetallic phases in the Ca–Al–Pt system

Peter C. Müller,[a] Linda S. Reitz,[a] Stefan Engel,[b] Richard Dronskowski,[a,*] and Oliver Janka[b,*]

[a] Institute of Inorganic Chemistry, RWTH Aachen University, 52056 Aachen, Germany

[b] Inorganic Solid State Chemistry, Saarland University, Campus C4.1, 66123 Saarbrücken, Germany

* **Corresponding authors: Richard Dronskowski,** Institute of Inorganic Chemistry, RWTH Aachen University, 52056 Aachen, Germany; Jülich-Aachen Research Alliance (JARA-CSD), RWTH Aachen University, 52056 Aachen, Germany; **Oliver Janka**, Inorganic Solid State Chemistry, Saarland University, Campus C4.1, 66123 Saarbrücken, Germany, e-mail: oliver.janka@uni-saarland.de

Keywords: intermetallics, crystal structure, covalent bonding, electronic structure

Table S1. Experimental (exp) and calculated (DFT) lattice parameters and unit cell volumes of Ca, Al and Pt.

compound	structure type	*a* (pm)	*V* (nm^3)	Ref.
Ca (exp)	Cu; $Fm\bar{3}m$	556	0.1712	[1]
Ca (DFT)		545.8	0.1626	*
Al (exp)	Cu; $Fm\bar{3}m$	405	0.0664	[2]
Al (DFT)		401.5	0.0647	*
Pt (exp)	Cu; $Fm\bar{3}m$	402	0.0650	[3]
Pt (DFT)		392.0	0.0602	*

* this work

Table S2. Experimental (exp) and calculated (DFT) lattice parameters and unit cell volumes of selected binary phases in the system Ca–Al, Ca–Pt and Al–Pt.

compound	structure type	*a* (pm)	*b* (pm)	*c* (pm)	*β* (pm)	*V* (nm^3)	Ref.
$CaAl_2$ (exp)	$MgCu_2$; $Fd\bar{3}m$	802.2	*a*	*a*	90	0.5162	[4]
$CaAl_2$ (DFT)		794.7	*a*	*a*	90	0.5020	*
$CaAl_4$ (exp)	$CaGa_4$; $C2/m$	615.26	617.3	632.9	118.03	0.2122	[4]
$CaAl_4$ (DFT)		608.3	609.2	635.7	118.27	0.2075	*
$CaPt_2$ (exp)	$MgCu_2$; $Fd\bar{3}m$	762.9	*a*	*a*	90	0.4440	[5]
$CaPt_2$ (DFT)		761.0	*a*	*a*	90	0.4406	*
$CaPt_5$ (exp)	$CaCu_5$; $P6/mmm$	532.2	*a*	436.8	90	0.1071	[6]
$CaPt_5$ (DFT)		532.0	*a*	437.2	90	0.1072	*
Al_2Pt (exp)	CaF_2; $Fm\bar{3}m$	591	*a*	*a*	90	0.2064	[7]
Al_2Pt (DFT)		589.7	*a*	*a*	90	0.2051	*
AlPt (exp)	FeSi; $P2_13$	487	*a*	*a*	90	0.1155	[8]
AlPt (DFT)		487.0	*a*	*a*	90	0.1155	*
Al_3Pt_2 (exp)	Al_3Ni_2; $P\bar{3}m1$	420.8	*a*	517.2	90	0.0793	[9]
Al_3Pt_2 (DFT)		420.6	*a*	518.6	90	0.0794	*

* this work

Table 3. Experimental (exp) and calculated (DFT) lattice parameters and unit cell volumes of all known phases in the ternary system Ca–Al–Pt.

compound	structure type	*a* (pm)	*b* (pm)	*c* (pm)	*β* (pm)	*V* (nm^3)	Ref.
CaAlPt (exp)	TiNiSi; *Pnma*	717.22	428.85	777.60	90	0.2392	[10]
CaAlPt (DFT)		712.96	424.46	776.61	90	0.2350	*
$CaAl_2Pt$ (exp)	$MgAl_2Cu$; *Cmcm*	418.82	1046.96	721.94	90	0.3166	[11]
$CaAl_2Pt$ (DFT)		418.19	1035.28	719.39	90	0.3115	*
$CaAl_2Pt_2$ (exp)	$CaBe_2Ge_2$; *P*4/*nmm*	426.79	*a*	988.79	90	0.1801	[12]
$CaAl_2Pt_2$ (DFT)		412.65	*a*	1072.50	90	0.1826	*
$CaAl_5Pt_3$ (exp)	YNi_5Si_3; *Pnma*	2050.3	409.0	736.4	90	0.6175	[13]
$CaAl_5Pt_3$ (DFT)		2045.97	409.00	734.74	90	0.6148	*
Ca_2AlPt_2 (exp)	Ca_2SiIr_2; *C*2/*c*	981.03	573.74	772.95	101.86	0.4258	[14]
Ca_2AlPt_2 (DFT)		977.91	568.67	770.08	102.11	0.4187	*
Ca_2Al_3Pt (exp)	Mg_2Cu_3Si; $P6_3/mmc$	561.46	*a*	876.94	90	0.2394	[12]
Ca_2Al_3Pt (DFT)		557.21	*a*	867.98	90	0.2339	*
$Ca_2Al_9Pt_3$ (exp)	$Y_2Co_3Ga_9$; *Cmcm*	1310.49	761.67	947.88	90	0.9461	[15]
$Ca_2Al_9Pt_3$ (DFT)		1308.81	760.46	945.06	90	0.9406	*
$Ca_2Al_{15}Pt_6$ (exp)	$Sc_2Al_{15}Pt_6$; $P2_1/m$	741.11	1665.70	741.11	119.99	0.7923	[16]
$Ca_2Al_{15}Pt_6$ (DFT)		739.27	1663.75	739.29	119.97	0.7877	*
$Ca_2Al_{16}Pt_9$ (exp)	$Ce_2Al_{16}Pt_9$; *Immm*	414.03	1181.77	442.12	90	0.8956	[17]
$Ca_2Al_{16}Pt_9$ (DFT)		413.15	1187.90	1826.88	90	0.8966	*

* this work

Table 4. Löwdin charge, bond count, interatomic distances as well as ICOBI values for Ca, Pt and Al.

compound	Löwdin charge	*d*(*M*–*M*) /pm	ICOBI(*M*–*M*)
Ca	±0.00	386	0.12
Pt	±0.00	277	0.09
Al	±0.00	284	0.23

Table 5. Löwdin charge, *ab initio* oxidation number (ON_{ai}), atom pair, bond count, interatomic distances as well as ICOBI values for $CaAl_2$, $CaAl_4$, $CaPt_2$, $CaPt_5$ and Al_2Pt, AlPt and Al_3Pt_2.

compound	atom	Löwdin charge	ON_{ai}	atom pair	distance /pm	ICOBI(M–M)
$CaAl_2$	Ca	+1.54	+1.86	Ca–Ca	344	0.01
				Ca–Al	329	0.05
	Al	–0.77	–0.93	Al–Al	281	0.43
$CaAl_4$	Ca	+1.66	+1.92	Ca–Al1	351	0.02
				Ca–Al1	355	0.02
				Ca–Al2	328	0.04
				Ca–Al2	330	0.04
				Ca–Al2	331	0.04
	Al1	–0.25	+.031	Al1–Al1	299	0.23
				Al1–Al1	304	0.22
				Al1–Al2	264	0.54
				Al1–Al2	265	0.52
	Al2	–0.60	–1.27	Al2–Al2	253	0.81
$CaPt_2$	Ca	+1.17	+1.52	Ca–Ca	329	0.03
				Ca–Pt	315	0.04
	Pt	–0.58	–0.76	Pt–Pt	269	0.11
$CaPt_5$	Ca	+1.44	+1.69	Ca–Ca	437	0.00
				Ca–Pt1	307	0.05
				Ca–Pt2	344	0.02
	Pt1	–0.38	–0.32	Pt1–Pt1	307	0.03
				Pt1–Pt2	267	0.13
	Pt2	–0.23	–0.35	Pt2–Pt2	266	0.13
Al_2Pt	Al	–0.13	+0.41	Al–Al	295	0.27
				Al–Pt	255	0.26
	Pt	+0.26	–0.82	Pt–Pt	417	0.02
AlPt	Al	–0.39	–0.05	Al–Al	301	0.18
				Al–Pt	260	0.25
				Al–Pt	261	0.25
				Al–Pt	261	0.17
	Pt	+0.39	+0.05	Pt–Pt	301	0.04
Al_3Pt_2	Al1	–0.18	+0.62	Al1–Al2	304	0.23
				Al1–Pt	250	0.30
	Al2	–0.29	–0.35	Al2–Al2	287	0.31
				Al2–Pt	257	0.22
				Al2–Pt	262	0.24
				Al2–Pt	268	0.15
	Pt	+0.32	–0.44	Pt–Pt	297	0.04

Table 6. Löwdin charge, atom pair, bond count, interatomic distances as well as ICOBI values for all known phases in the ternary system Ca–Al–Pt. Important distances are highlighted in bold.

compound	atom	Löwdin charge	ON_{ai}	atom pair	distance /pm	ICOBI(*M*–*M*)
CaAlPt	Ca	+1.26	+1.66	Ca–Ca	347	0.01
				Ca–Al	314	0.04
				Ca–Pt	296	0.05
	Al	–1.08	–0.65	Al–Al	311	0.26
				Al–Pt	257	0.29
				Al–Pt	260	0.33
				Al–Pt	266	0.43
	Pt	–0.17	–1.01	Pt–Pt	410	0.02
$CaAl_2Pt$	Ca	+1.47	+1.81	Ca–Ca	381	0.00
				Ca–Al	320	0.05
				Ca–Pt	287	0.04
	Al	–0.84	–0.82	Al–Al	279	0.42
				Al–Al	292	0.34
				**Al–Al**	**340**	**0.31**
				Al–Pt	255	0.32
				Al–Pt	258	0.29
	Pt	+0.22	–0.18	Pt–Pt	>500	–
$CaAl_2Pt_2$	Ca	+1.46	+1.82	Ca–Ca	413	0.00
				Ca–Al2	316	0.03
				Ca–Al1	318	0.07
				Ca–Pt2	338	0.01
				Ca–Pt1	338	0.03
	Al1	–0.66	–0.36	Al1–Al1	292	0.30
				Al1–Pt1	253	0.28
	Al2	–1.06	–1.03	Al2–Pt1	250	0.45
				Al2–Pt2	250	0.34
	Pt1	+0.06	–0.39	Pt1–Pt1	413	0.01
	Pt2	+0.20	–0.05	Pt2–Pt2	292	0.08

compound	atom	Löwdin charge	ON_{ai}	atom pair	distance /pm	ICOBI(*M*–*M*)
$CaAl_5Pt_3$	Ca	+1.49	+1.84	Ca–Ca	409	0.00
				Ca–Al5	317	0.03
				Ca–Pt3	320	0.03
	Al1	–0.43	+0.09	Al1–Al1	281	0.35
				Al1–Al4	306	0.22
				Al1–Al5	278	0.32
				Al1–Pt1	253	0.37
				Al1–Pt2	261	0.32
				Al1–Pt2	265	0.27
				Al1–Pt2	266	0.28
	Al2	–0.56	–0.40	Al2–Al3	283	0.31
				Al2–Al3	289	0.32
				Al2–Al4	293	0.28
				Al2–Pt1	261	0.32
				Al2–Pt3	256	0.31
				Al2–Pt3	262	0.22
	Al3	–0.55	–0.42	Al3–Al4	286	0.27
				Al3–Pt2	254	0.19
				Al3–Pt3	250	0.32
				Al3–Pt3	256	0.25
	Al4	–0.38	–0.36	Al4–Al5	282	0.34
				Al4–Pt1	264	0.23
				Al4–Pt2	272	0.18
				Al4–Pt3	250	0.25
	Al5	–0.40	–0.54	Al5–Al5	275	0.27
				Al5–Pt1	246	0.24
				Al5–Pt2	253	0.22
	Pt1	+0.28	–0.31	Pt1–Pt2	306	0.02
	Pt2	+0.28	–0.29	Pt2–Pt2	407	0.02
	Pt3	+0.26	–0.32	Pt3–Pt3	409	0.02
Ca_2AlPt_2	Ca	+1.16	+1.56	Ca–Ca	357	0.01
				Ca–Al	326	0.07
				Ca–Pt	304	0.02
	Al	–1.43	–1.23	Al–Pt	250	0.36
				Al–Pt	252	0.36
	Pt	–0.45	–0.94	**Pt–Pt**	**270**	**0.30**
				**Pt–Pt**	**304**	**0.03**
Ca_2Al_3Pt	Ca	+1.44	+1.78	Ca–Ca	336	0.01
				Ca–Al	313	0.05
				Ca–Pt	325	0.03
	Al	–1.03	–1.07	Al–Al	272	0.37
				**Al–Al**	**285**	**0.52**
				Al–Pt	268	0.28
	Pt	+0.23	–0.37	Pt–Al	434	0.04

compound	atom	Löwdin charge	ON_{ai}	atom pair	distance /pm	ICOBI(*M*–*M*)
$Ca_2Al_9Pt_3$	Ca	+1.62	+1.89	Ca–Ca	427	0.01
				Ca–Al	303	0.03
				Ca–Pt	343	0.02
	Al1	–0.59	–0.84	Al1–Al1	289	0.34
				Al1–Al1	292	0.32
				Al1-Al2	280	0.31
				Al1-Al3	280	0.33
				Al1-Al4	275	0.33
	Al2	–0.40	+0.13	Al2–Al2	269	0.37
				Al2–Al3	284	0.33
				Al2–Al4	274	0.35
	Al3	–0.49	–0.21	Al3–Al3	274	0.35
	Al4	–0.44	–0.06	–	–	–
	Pt1	+0.45	–0.06	Pt1–Al1	257	0.30
				Pt1–Al1	263	0.22
				Pt1–Al2	263	0.20
				Pt1–Al3	256	0.26
	Pt2	+0.44	–0.06	Pt2–Al1	264	0.22
				Pt2–Al3	258	0.26
				Pt2–Al4	256	0.26
$Ca_2Al_{15}Pt_6$	Ca	+1.59	+1.88	–	–	–
	Al1	–0.48	–0.43	Al1–Al1	264	0.43
				Al1–Al2	281	0.30
				Al1–Al3	286	0.32
				Al1–Pt	257	0.26
	Al2	–0.45	–0.60	Al2–Al3	281	0.30
				Al2–Al2	289	0.30
				Al2–Pt	250	0.26
	Al3	–0.23	+0.57	Al3–Al3	289	0.30
				Al3–Pt	250	0.26
				Al3–Pt	256	0.26
	Pt	+0.38	–0.36	–	–	–

compound	atom	Löwdin charge	ON_{ai}	atom pair	distance /pm	ICOBI(*M*–*M*)
$Ca_2Al_{16}Pt_9$	Ca	+1.55	+1.87	Ca–Ca	413	0.00
				Ca–Al	330	0.04
				Ca–Pt	328	0.03
	Al1	–0.36	–0.13	Al1–Al1	282	0.34
				Al1–Al2	329	0.13
				Al1–Al3	286	0.27
				Al1–Al5	274	0.36
				Al1–Pt1	248	0.43
				Al1–Pt2	252	0.26
				Al1–Pt3	253	0.27
	Al2	–0.35	–0.42	Al2–Al2	294	0.24
				Al2–Al3	281	0.31
				Al2–Al4	289	0.31
				Al2–Al5	294	0.19
				Al2–Pt1	253	0.26
				Al2–Pt2	271	0.25
				Al2–Pt3	269	0.18
				Al2–Pt4	250	0.25
	Al3	–0.48	–0.10	Al3–Al3	299	0.29
				Al3–Pt1	250	0.21
				Al3-Pt1	266	0.19
	Al4	–0.42	–0.04	Al4–Al4	319	0.17
				Al4–Pt1	257	0.31
				Al4–Pt4	261	0.21
	Al5	–0.20	+0.75	Al5–Al5	285	0.27
				Al5–Pt2	264	0.19
				Al5–Pt3	263	0.24
	Pt1	+0.28	–0.24	Pt1–Pt3	300	0.03
	Pt2	+0.35	–0.27	–	–	–
	Pt3	+0.39	–0.40	–	–	–
	Pt4	+0.32	–0.25	–	–	–

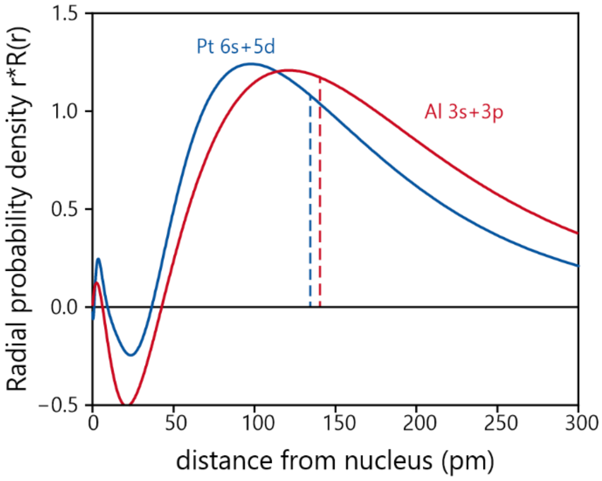

Figure S1. Cumulative radial probability density of the valence orbitals for Pt and Al. Note that values of Al are significantly higher at larger distances, enabling better overlap at longer Al–Al distances.

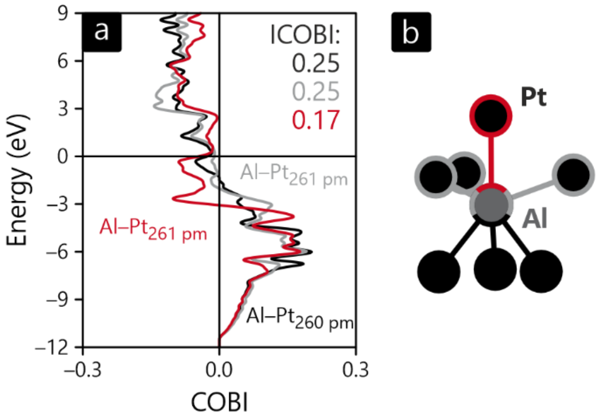

Figure S2. a) Energy-dependent crystal orbital bond index of the Al–Pt bonds in AlPt. b) Polyhedron of the nearest neighbors for Al. Despite similar lengths for all bonds in this polyhedron, the respective ICOBI differ significantly.

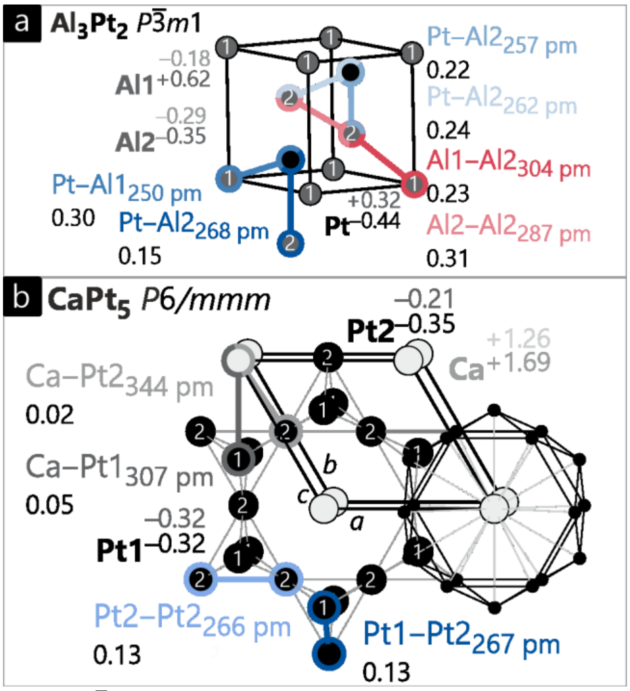

Figure S3. Unit cell of a) Al_3Pt_2 ($P\bar{3}m1$) and b) $CaPt_9$ (P6/*mmm*). Ca atoms are depicted in light grey, Al atoms in grey and Pt atoms in black. The Löwdin charges and oxidation numbers are given in addition to interatomic distances and respective ICOBI values for the different bonds.

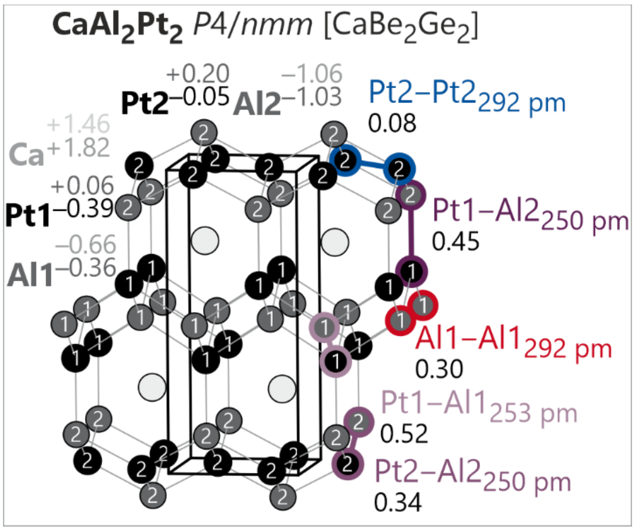

Figure S4. Unit cell of $CaAl_2Pt_2$ ($CaBe_2Ge_2$ type, *P4/nmm*). Ca atoms are depicted in light grey, Al atoms in grey and Pt atoms in black. The Löwdin charges and oxidation numbers are given in addition to interatomic distances and respective ICOBI values for the different bonds.

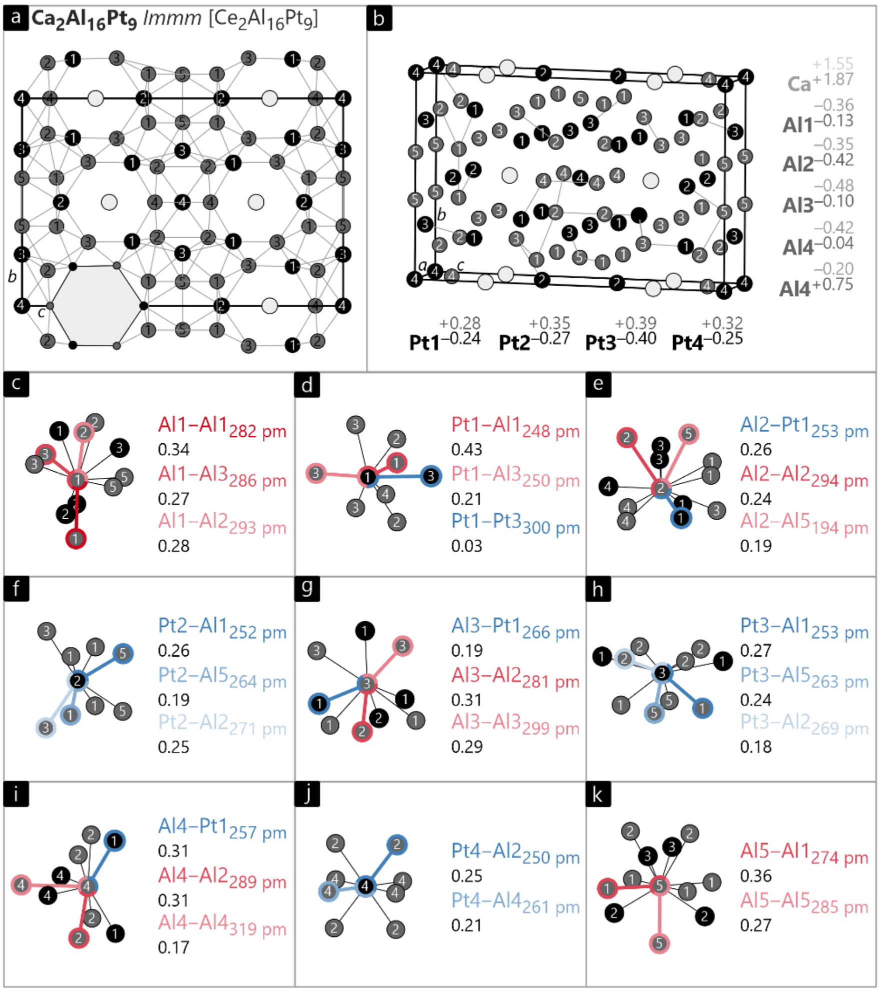

Figure S5. a), b) Unit cell of $Ca_2Al_{16}Pt_9$ ($Ce_2Al_{16}Pt_9$ type, *Immm*). Ca atoms are depicted in light grey, Al atoms in grey and Pt atoms in black. The Löwdin charges and oxidation numbers are given in b). c)–k) Interatomic distances and respective ICOBI values for the different bonds are indicated.

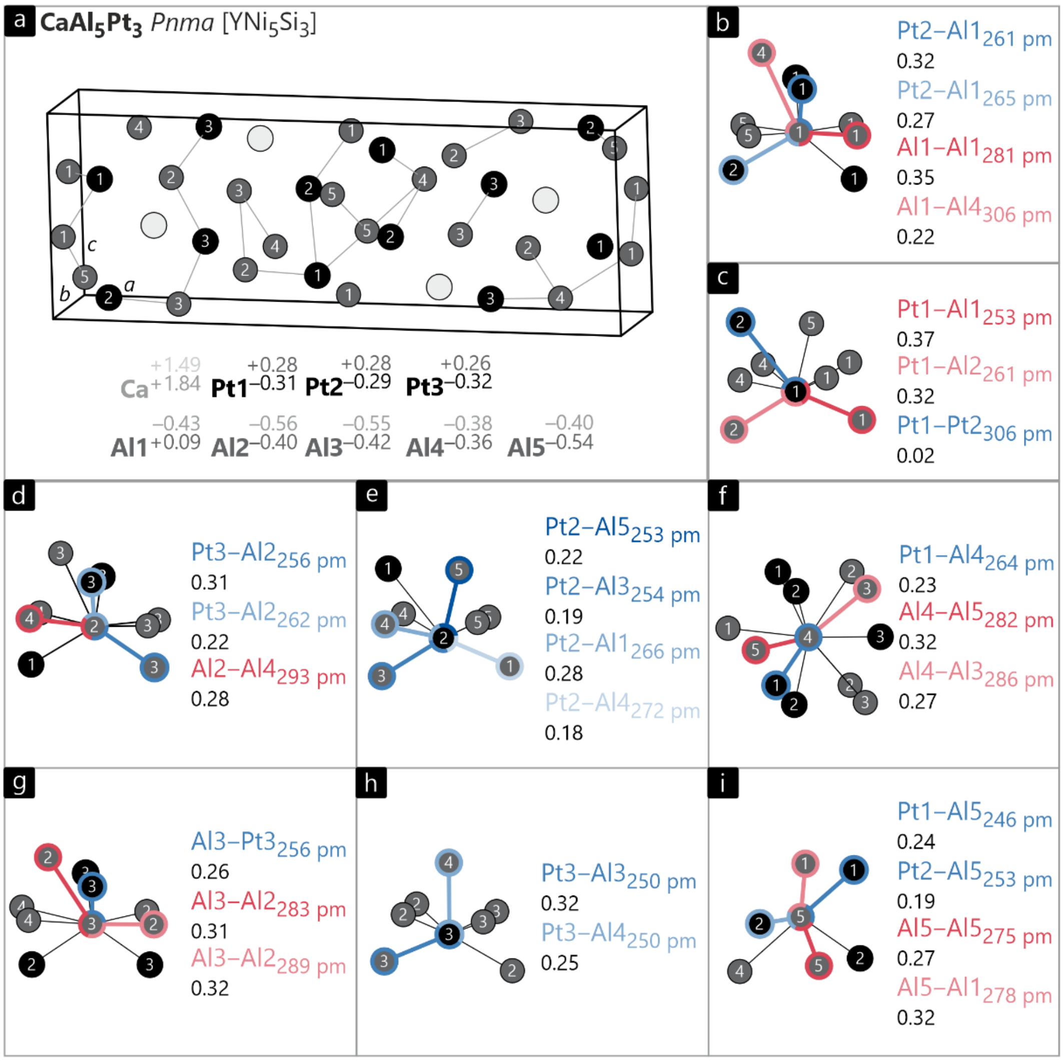

Figure S6. a) Unit cell of orthorhombic $CaAl_5Pt_3$ with Löwdin charges and *ab initio* oxidation numbers. b)–i) Interatomic distances and respective ICOBI values for the different bonds are indicated.

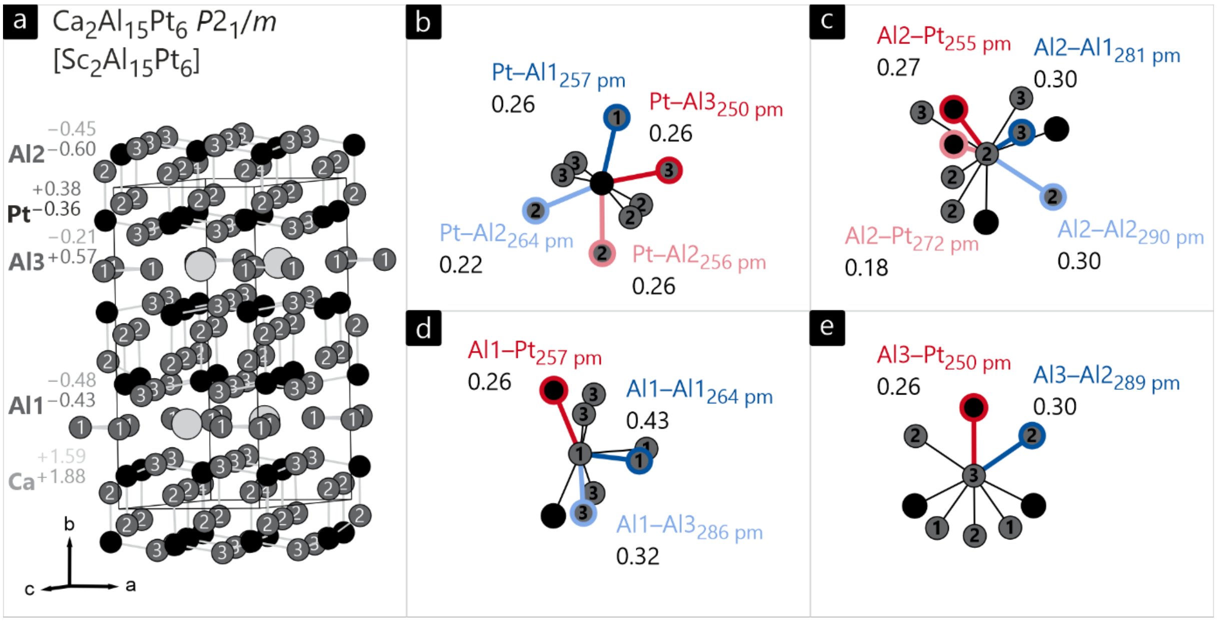

Figure S7. a) Unit cell of orthorhombic $Ca_2Al_{15}Pt_6$. Oxidation numbers and Löwdin charges are given as superscript for the respective atoms. b-e) Interatomic distances and respective ICOBI values for the different polyhedra.

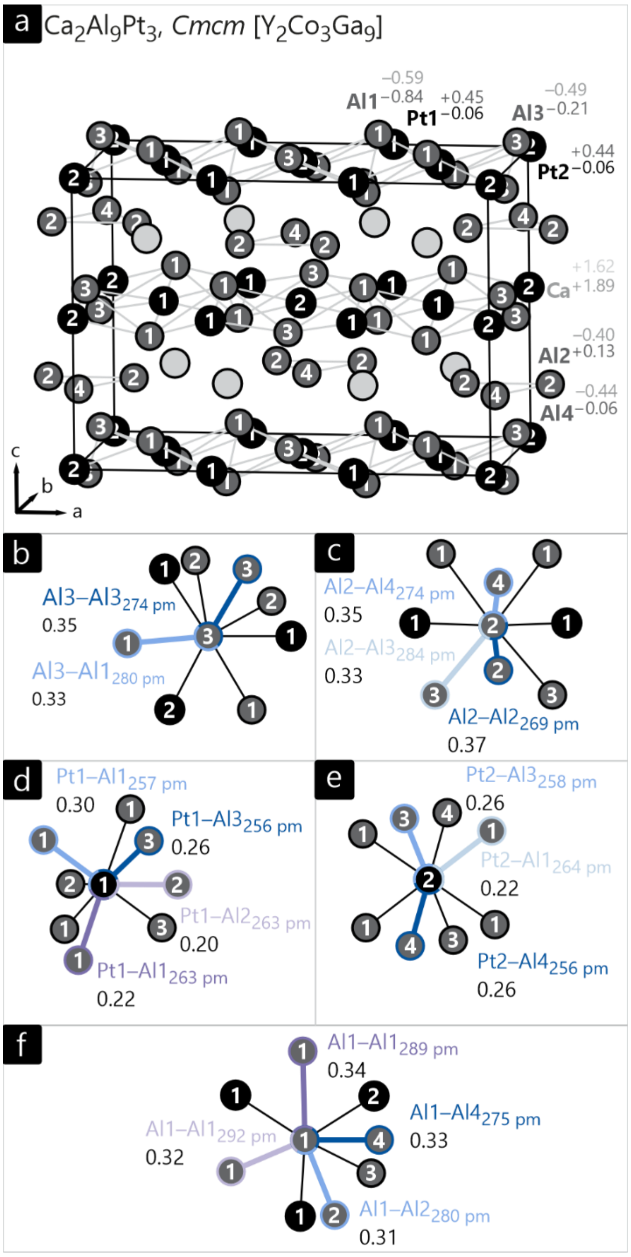

Figure S8. a) Unit cell of orthorhombic $Ca_2Al_9Pt_3$ with Löwdin charges and *ab initio* oxidation numbers. b)–f) Interatomic distances and respective ICOBI values for the different bonds are indicated.

References

(1) Hull, A. W., The Arrangement of Atoms in Some Common Metals. *Science* **1920**, *52*, 227–229.

(2) Hull, A. W., A new method of X-ray crystal analysis. *Phys. Rev.* **1917**, *10*, 661–696.

(3) Hull, A. W., The positions of atoms in metals. *Trans. Am. Inst. Electro. Eng.* **1919**, *38*, 1445–1466.

(4) Engel, S.; Gießelmann, E. C. J.; Schank, L. E.; Heymann, G.; Brix, K.; Kautenburger, R.; Beck, H. P.; Janka, O., Theoretical and ^{27}Al NMR spectroscopic investigations of binary intermetallic alkaline-earth aluminides. *Inorg. Chem.* **2023**, *62*, 4260–4271.

(5) Wood, E. A.; Compton, V. B., Laves-phase compounds of alkaline earths and noble metals. *Acta Crystallogr.* **1958**, *11*, 429–433.

(6) Bronger, W.; Klemm, W., Darstellung von Legierungen des Platins mit unedlen Metallen. *Z. Anorg. Allg. Chem.* **1962**, *319*, 58–81.

(7) Zintl, E.; Harder, A.; Haucke, W., Legierungsphasen mit Fluoritstruktur (22. Mitteilung über Metalle und Legierungen). *Z. Phys. Chem. B* **1937**, *35*, 354–362.

(8) Schubert, K.; Burkhardt, W.; Esslinger, P.; Günzel, E.; Meissner, H. G.; Schütt, W.; Wegst, J.; Wilkens, M., Einige strukturelle Ergebnisse an metallischen Phasen. *Naturwissenschaften* **1956**, *43*, 248–249.

(9) Ferro, R.; Capelli, R.; Rambaldi, G.; Bonino, G. B., Ricerche sulle leghe dei metalli nobili con gli elementi piu elettropositivi. VI. Esame micrografico e roentgenografico di alcune leghe del sistema alluminio-platino. *Atti Accad. Naz. Lincei, Cl. Sci. Fis., Mat. Nat., Rend.* **1963**, *34*, 45–47.

(10) Hulliger, F., On new ternary aluminides *Ln*PdAl and *Ln*PtAl. *J. Alloys Compd.* **1993**, *196*, 225–228.

(11) Stegemann, F.; Block, T.; Klenner, S.; Zhang, Y.; Fokwa, B. P. T.; Timmer, A.; Mönig, H.; Doerenkamp, C.; Eckert, H.; Janka, O., From 3D to 2D: structural, spectroscopical and theoretical investigations of the dimensionality reduction in the $[PtAl_2]^{\delta-}$ polyanions of the isotypic MPtAl$_2$ series (M = Ca–Ba, Eu). *Chem. Eur. J.* **2019**, *25*, 10735–10747.

(12) Engel, S.; Koch, M.; Janka, O., Nominal $CaAl_2Pt_2$ and Ca_2Al_3Pt – two new Intermetallic Compounds in the Ternary System Ca−Al−Pt. *Z. Anorg. Allg. Chem.* **2024**, *650*, e202400094.

(13) Engel, S.; Gießelmann, E. C. J.; Schumacher, L.; Zhang, Y.; Müller, F.; Janka, O., Synthesis, magnetic and NMR spectroscopic properties of the MAl$_5$Pt$_3$ series (M = Ca, Y, La-Nd, Sm-Er). *Dalton Trans.* **2024**, *53*, 12176–12188.

(14) Doverbratt, I.; Ponou, S.; Zhang, Y.; Lidin, S.; Miller, G. J., Linear Metal Chains in Ca_2M_2X (M = Pd, Pt; X = Al, Ge): Origin of the Pairwise Distortion and Its Role in the Structure Stability. *Chem. Mater.* **2015**, *27*, 304–315.

(15) Stegemann, F.; Zhang, Y.; Fokwa, B. P. T.; Janka, O., On the formation of the $Gd_3Ru_4Al_{12}$ versus the $Y_2Co_3Ga_9$ type structure – $M_3Rh_4Al_{12}$ (M = Ca, Eu) versus $M_2T_3Al_9$ (M = Ca, Sr, Eu, Yb; T = Ir, Pt). *Dalton Trans.* **2020**, *49*, 6398–6406.
(16) Radzieowski, M.; Stegemann, F.; Hoffmann, R.-D.; Janka, O., The monoclinic superstructure of the $M_2Pt_6Al_{15}$ series (M = Ca, Sc, Y, La, Lu). *Z. Kristallogr.* **2017**, *232*, 675–687.
(17) Engel, S.; Janka, O., New alkaline earth and rare earth representatives adopting the $Ce_2Al_{16}Pt_9$ type structure. *Z. Naturforsch.* **2024**, *79b*, 595–603.
